# Phenotypic and molecular characterization of *Salmonella enterica* isolated from retail beef in Peshawar, Pakistan

**DOI:** 10.1371/journal.pone.0352859

**Published:** 2026-07-29

**Authors:** Muhammad Jawad Ullah, Kafeel Ahmad, Fawad Inayat, Faryal Khattak, Sawaira Huriya, Fai Sal

**Affiliations:** 1 Centre of Biotechnology and Microbiology, University of Peshawar, Peshawar, Khyber Pakhtunkhwa, Pakistan; 2 Department of Biochemistry, Abdul Wali Khan University, Mardan, Khyber Pakhtunkhwa, Pakistan; 3 Department of Allied Health Sciences, Kabul Medical University, Kabul, Afghanistan; Tribhuvan University, NEPAL

## Abstract

*Salmonella enterica* is a major foodborne pathogen associated with beef contamination and increasing antimicrobial resistance, particularly in low- and middle-income countries. This study investigated the prevalence, antimicrobial resistance patterns, and genetic diversity of *S. enterica* isolated from retail beef samples in Peshawar, Pakistan, using combined phenotypic and molecular approaches. A total of 250 beef samples were collected from urban, rural, and peri-urban retail markets. *Salmonella enterica* was isolated using standard culture and biochemical methods and confirmed by PCR targeting the *invA* gene. Antimicrobial susceptibility was determined by the Kirby–Bauer disk diffusion method, and genetic diversity was assessed using randomly amplified polymorphic DNA PCR (RAPD-PCR). Overall, *S. enterica* was detected in 150 samples (60.0%), with higher prevalence in rural markets (77.5%) compared with urban (52.5%) and peri-urban (50.0%) locations. A high percentage of isolates were resistant to azithromycin (94.0%), tetracycline (68.7%), and streptomycin (52.0%), indicating widespread multidrug resistance among isolates. Five isolates (3.3%) were identified as extended-spectrum β-lactamase (ESBL) producers and carried *bla*TEM-1, *bla*CTX-M-15, or *bla*SHV-12 genes. RAPD-PCR analysis revealed substantial genetic heterogeneity, with isolates distributed across multiple phylogenetic clusters, suggesting diverse contamination sources rather than clonal dissemination. These findings indicate that retail beef in Peshawar represents a significant reservoir of genetically diverse and antimicrobial-resistant *S. enterica*. The combined use of phenotypic methods with targeted molecular and low-cost genotyping approaches provides a practical framework for surveillance of foodborne *Salmonella* in resource-limited settings.

## Introduction

*Salmonella* remains one of the most significant foodborne pathogens worldwide. It poses severe public health risks and economic burdens due to outbreaks linked to contaminated animal products, particularly beef [1]. The genus *Salmonella* comprises over 2,600 serovars. Many of these serovars are zoonotic and are associated with gastroenteritis, systemic infections, and mortality in immunocompromised individuals [2]. Beef is a significant source of dietary protein. Consequently, it has the potential to serve as a vehicle for the transmission of *Salmonella*. Contamination is most likely when hygiene standards are suboptimal or when cattle are subclinically infected [3,4]. The pathogen is highly adaptable and genetically diverse. Therefore, understanding the phylogeny and genetic diversity of *Salmonella* is essential for pathogen tracking, source attribution, and antimicrobial resistance (AMR) surveillance [5].

Conventional microbiological techniques form the foundation of *Salmonella* detection. These methods rely on selective growth, biochemical analysis, and serotyping [6]. Although they are useful for preliminary identification, these techniques have limited discriminatory power. They often cannot distinguish between closely related strains. Furthermore, they may fail to identify emerging variants with atypical metabolic patterns [7,8]. To overcome these limitations, molecular analysis is required for robust and conclusive characterization of bacterial strains. Phenotypic approaches alone cannot reliably identify virulence and AMR genotypes.

Molecular examination of conserved virulence genes has proven to be a key tool for rapid and accurate pathogen assessment. The type III secretion system (T3SS) of *Salmonella* is used as a genetic marker. Specifically, the *invA* gene is found across diverse *Salmonella* serovars [9]. PCR-based detection of *invA* is species-specific. It improves diagnostic performance in complex matrices such as beef [10,11]. However, *invA* PCR can only confirm the presence of the pathogen. It does not provide strain-level discriminatory information. Therefore, higher-resolution genotyping is necessary to obtain meaningful epidemiological data.

Random amplified polymorphic DNA-PCR (RAPD-PCR) is a rapid and inexpensive method for evaluating genetic dissimilarity among bacterial isolates [12]. Unlike conventional serotyping, RAPD-PCR produces strain-specific banding patterns. This is achieved by amplifying random genomic regions using short, arbitrary primers. Phylogenetic clustering of strains is possible without prior sequence information [13]. This technique has been used effectively to track *Salmonella* outbreaks and differentiate closely related strains in foodborne isolates [14]. For laboratories with limited resources, RAPD-PCR offers a viable alternative to whole genome sequencing (WGS). It balances lower implementation costs with adequate resolution for initial screening.

Although substantial research has focused on *Salmonella* in poultry and swine, less is known about beef-associated strains. There is a specific gap in knowledge regarding the genetic diversity of beef-derived isolates and their associated transmission risks. Phenotypic methods provide initial identification but lack the discriminatory power needed for strain-level analysis. This study addresses these gaps by combining phenotypic analysis with molecular tools to characterize *Salmonella* isolates from beef. The specific objectives were to: (1) isolate and phenotypically identify *Salmonella* from beef samples, (2) confirm isolates through *invA* gene detection, and (3) analyze genetic diversity and phylogenetic relationships using RAPD-PCR and Nei and Li coefficients. This integrated approach has the potential to enhance the understanding of beef-associated *Salmonella* strains and support improved food safety interventions.

## Materials and methods

### Sample collection

A total of 250 fresh beef samples were aseptically collected from randomly selected retail outlets in Peshawar, Pakistan, from 01 February 2021–30 January 2023. A sample size of 250 was determined based on a convenience sampling strategy, aiming to capture diversity across the three distinct retail environments (urban, rural, and peri-urban) within the constraints of time and laboratory resources. This sample size is comparable to or larger than similar regional surveillance studies for foodborne pathogens [15]. Samples (approximately 25 g each) were obtained via sterile forceps and placed in pre-labelled sterile sampling bags to maintain integrity. To ensure representative sampling, cuts were taken from both the superficial and deep muscle layers. The samples were immediately transported to the Laboratory of Food Microbiology at the Centre of Biotechnology and Microbiology, University of Peshawar, in insulated coolers maintained at 4 °C and processed within 2 hours of collection to minimize microbial shifts [16].

Each sample was homogenized in 225 mL of buffered peptone water in a sterile flask. The sample was incubated at 37 °C for 24 h for pre-enrichment. The enriched cultures were streaked onto the selective media: xylose lysine deoxycholate (XLD) agar and *Salmonella*-Shigella (SS) agar, followed by incubation at 37 °C for 24 h. Presumptive *Salmonella* colonies (black centres on XLD due to H_2_S production) were subcultured onto SS agar. Gram staining confirmed the presence of Gram-negative, rod-shaped bacteria. Biochemical profiling included catalase test, oxidase test, triple sugar iron (TSI) agar, citrate utilization, indole, urease, and motility tests as reported previously [17].

### Quality control

For all culture and biochemical identification procedures, *Salmonella enterica* serovar Typhimurium ATCC 14028 was used as a positive control, and *Escherichia coli* ATCC 25922 was used as a negative control. For antimicrobial susceptibility testing, *E. coli* ATCC 25922 and *S. enterica* ATCC 14028 were included as quality-control strains in accordance with CLSI guidelines [18]. All PCR assays included a no-template control (NTC) to rule out reagent contamination.

### Antibiotic sensitivity and extended-spectrum beta-lactamase test

Antibiotic susceptibility of all confirmed *Salmonella enterica* isolates (n = 150) was performed using the Kirby–Bauer disk diffusion method on Mueller-Hinton agar (MHA) based on the guidelines of the Clinical and Laboratory Standards Institute (CLSI M100) [18]. Bacterial suspensions were standardized to a 0.5 McFarland turbidity (~1.5 × 10⁸ CFU/mL) and swabbed on MHA plates. A panel of 16 antibiotic discs (Oxoid, UK) representing 10 classes of antimicrobials was placed on the inoculated agar aseptically. The plates were incubated at 37 °C for 16–18 h.

Zone diameters were measured to the nearest mm. Isolates were classified as susceptible, intermediate, or resistant according to CLSI interpretive criteria. Multidrug resistance (MDR) was defined as acquired non-susceptibility to at least one agent in three or more antimicrobial categories [19]. From the assembled data, the prevalence of resistance to each antibiotic was calculated.

For ESBL screening, a phenotypic confirmatory double-disk synergy test was performed in accordance with CLSI M100 guidelines [18]. A bacterial suspension adjusted to a 0.5 McFarland standard was lawn-cultured onto an MHA plate. An amoxicillin-clavulanate (20/10 μg) disc was placed at the centre of the plate, and ceftazidime (30 μg), cefotaxime (30 μg), and ceftriaxone (30 μg) discs were placed 20 mm (centre to centre) from the amoxicillin-clavulanate disc. The plates were incubated at 37 °C for 16–18 h. Enhancement of the zone of inhibition of any cephalosporin disc towards the amoxicillin-clavulanate disc was interpreted as a positive result and confirmed the isolate as an ESBL producer.

### Molecular identification

DNA was extracted from pure cultures using the GeneJET Genomic DNA Purification Kit (Thermo Scientific, USA). The *invA* gene (284 bp), a *Salmonella*-specific marker, was amplified using the forward primer invA-F: 5′-GTGAAATTATCGCCACGTTCGGGCAA-3′ and reverse primer invA-R: 5′-TCATCGCACCGTCAAAGGAACC-3′ [20]. The PCR conditions were as follows: 95 °C for 5 min (initial denaturation); 35 cycles of 95 °C for 30 s, 55 °C for 30 s, and 72 °C for 30 s; and a final extension at 72 °C for 5 min. Amplicons were electrophoresed on a 1.5% agarose gel (100 V, 45 min) and visualized under UV light. A 100 bp DNA ladder was used for size comparison.

For molecular confirmation of ESBL, two genes, *bla*TEM and *bla*CTX-M, were amplified. Primers for *bla*TEM were forward 5′-ATGAGTATTCAACATTTCCGC-3′ and reverse 5′-CAATGCTTAATCAGTGAGG-3′ [21]. Primers for *bla*CTX-M were forward 5′-CGCTTTGCGATGTGCAG-3′ and reverse 5′-ACCGCGATATCGTTGGT-3′ [22]. A subset of *bla*CTX-M-positive amplicons were subjected to Sanger sequencing and confirmed to contain the *bla*CTX-M-15 variant. The PCR conditions for both genes were as follows: 95 °C for 5 min (initial denaturation); 30 cycles of 94 °C for 1 min, 55 °C for 1 min, and 72 °C for 1 min; and a final extension at 72 °C for 7 min. Additionally, the presence of *bla*SHV genes was screened using previously described primers and conditions [23].

### Genetic diversity analysis

RAPD-PCR was performed using the primer OPS-11 (5′-CAGGCCCTTC-3′) [24,25]. The 20 μL reaction mixture contained 4 μL of 5 × FIREPol master mix, 1 μL of primer (10 μM), 1 μL of template DNA (50 ng/μL), and 14 μL of nuclease-free water. The cycling conditions were as follows: 95 °C for 5 min; 35 cycles of 94 °C for 1 min, 53 °C for 2 min, and 72 °C for 3 min; and a final extension at 72 °C for 7 min. Amplicons were separated on a 1.5% agarose gel (100 V, 35 min). The primer OPS-11 was selected based on its established utility for RAPD-based typing of *Salmonella* isolates in previous studies [26].

### Phylogenetic and statistical analyses

RAPD banding patterns were scored as binary data (1 = presence, 0 = absence). Genetic similarity was calculated using the Nei and Li coefficient [27], and dendrograms were constructed using the unweighted pair group method with arithmetic mean (UPGMA) in MEGA 11 [28]. The genetic distances between the ESBL-associated clones and all other clones were compared using a non-parametric test (Mann–Whitney U test) owing to the non-normal distribution of the pairwise distance data. A Bonferroni correction was applied to adjust for multiple comparisons where appropriate. Grubbs’ test was performed on the matrix of pairwise genetic distances to identify any possible genetic outliers within the ESBL-positive group. The co-occurrence of the *bla*TEM and *bla*CTX-M resistance genes among isolates was assessed using a Chi-square test. Fisher’s exact test was used instead of the Chi-square test when expected cell counts in contingency tables were less than five. This test evaluated associations between membership in ESBL-associated phylogenetic clones and specific multidrug-resistant phenotypes.

## Results

### Distribution of Salmonella enterica isolates

**Fig 1:**
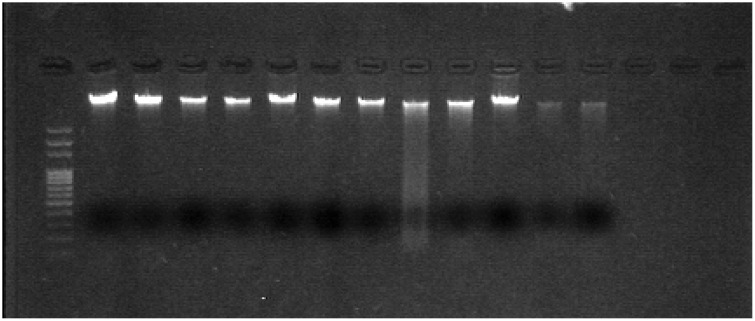
Agarose gel electrophoresis of extracted genomic DNA from representative *Salmonella enterica* isolates. Lane M contains a 100 bp DNA ladder. Lanes 1–15 contain genomic DNA extracted from 15 representative isolates. The presence of intact, high-molecular-weight bands confirms successful DNA extraction suitable for subsequent PCR-based assays.

A total of 250 fresh beef samples were collected from retail outlets across urban, rural, and peri-urban areas of the Peshawar district, Pakistan, to ensure representative geographic coverage. Among these samples, 150 (60.0%) were confirmed positive for *Salmonella enterica* through phenotypic and molecular detection methods. The highest prevalence was observed in rural areas, where 62 of 80 samples (77.5%) contained *Salmonella enterica*, followed by urban areas, with 63 positives of 120 samples (52.5%), and peri-urban areas, with 25 positives of 50 samples (50.0%) (Table 1 and Fig 1).

**Table 1:** Distribution of *Salmonella enterica* isolates from beef samples across Peshawar district.

Region type	No. of samples collected	No. of S. enterica isolates	Prevalence (%)
Urban areas	120	63	52.5
Rural areas	80	62	77.5
Peri-urban areas	50	25	50.0
**Total**	**250**	**150**	**60.0**

### Molecular confirmation of Salmonella enterica isolates

**Fig 2:**
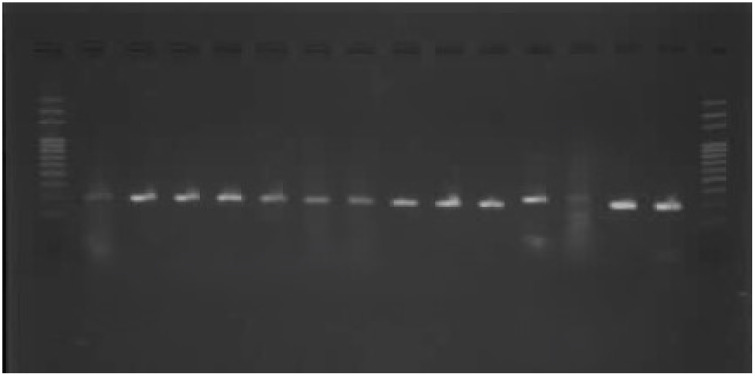
PCR amplification of the *invA* gene for species-level confirmation of *Salmonella enterica.* Lane M contains a 100 bp DNA ladder. Lanes 1–15 show PCR products from representative isolates. The expected amplicon size of 284 bp confirms the identity of the isolates as *Salmonella enterica*.

All phenotypically identified isolates were subjected to molecular confirmation by PCR amplification of the species-specific *invA* gene. The expected 284 bp amplicon was successfully detected in all 150 isolates, confirming their identity as *Salmonella enterica*. A representative gel image showing *invA* amplification is presented in Fig 1 and Fig 2.

### Antimicrobial susceptibility of the Salmonella enterica isolates

Table 2 summarizes the resistance profile of *Salmonella enterica* isolates against commonly used antibiotics. A high percentage of isolates were resistant to azithromycin (94.0%), tetracycline (68.7%), and streptomycin (52.0%), indicating widespread multidrug resistance. Moderate percentages of resistant isolates were noted for ampicillin (30.0%) and chloramphenicol (24.0%), while third- and fourth-generation cephalosporins (ceftriaxone, ceftazidime, cefepime) showed lower percentages of resistant isolates (10.7–12.0%). Fluoroquinolones such as ciprofloxacin and norfloxacin displayed comparatively low percentages of resistant isolates (6.0% and 2.7%, respectively), and carbapenem (imipenem) resistance remained rare (5.3%). These findings highlight significant public health risks posed by beef-derived *S. enterica*, with resistance concentrated in older and widely used antibiotic classes.

**Table 2:** Frequency and percentage of resistant isolates against various antibiotics (N = 150).

S. No.	Antibiotic	Disc code	Antibiotic class	Resistant isolates	%
1	Ampicillin	AMP	Penicillin	45	30.0
2	Azithromycin	AZM	Macrolide	141	94.0
3	Ceftriaxone	CRO	Cephalosporin	16	10.7
4	Ceftazidime	CAZ	Cephalosporin	17	11.3
5	Cefepime	FEP	Cephalosporin	18	12.0
6	Ciprofloxacin	CIP	Fluoroquinolone	9	6.0
7	Norfloxacin	NOR	Fluoroquinolone	4	2.7
8	Streptomycin	S	Aminoglycoside	78	52.0
9	Gentamicin	CN	Aminoglycoside	3	2.0
10	Amikacin	AK	Aminoglycoside	3	2.0
11	Sulfisoxazole	SCF	Sulfonamide	1	0.7
12	Trimethoprim-Sulfamethoxazole	SXT	Sulfonamide	31	20.7
13	Tetracycline	TE	Tetracycline	103	68.7
14	Imipenem	IPM	Carbapenem	8	5.3
15	Ofloxacin	OFX	Quinolone	12	8.0
16	Chloramphenicol	C	Chloramphenicol	36	24.0

### Analysis of extended-spectrum β-lactamase (ESBL) production

Among the 150 *Salmonella enterica* isolates, five (3.3%) were confirmed as ESBL producers through phenotypic screening using a double-disc synergy test in accordance with CLSI M100 guidelines (Table 3). All ESBL-positive isolates were resistant to β-lactam antibiotics, including ampicillin, amoxicillin-clavulanate, ceftriaxone, and ceftazidime, with resistance extending to other antibiotic classes, such as tetracyclines, fluoroquinolones, aminoglycosides, and sulfonamides, in various combinations.

**Table 3:** Antimicrobial resistance profile of ESBL-producing isolates (n = 5) compared to the total population (n = 150).

Antibiotic (disc potency)	Resistance in total isolates (%) (n = 150)	Resistance in ESBL producers (%) (n = 5)
Ampicillin (10 μg)	30.0%	100% (5/5)
Amoxicillin-Clavulanate (30 μg)	Not tested in total population	100% (5/5)
Ceftriaxone (30 μg)	10.7%	100% (5/5)
Ceftazidime (30 μg)	11.3%	100% (5/5)
Ciprofloxacin (5 μg)	6.0%	60% (3/5)
Tetracycline (30 μg)	68.7%	80% (4/5)
Trimethoprim-Sulfamethoxazole (25 μg)	20.7%	60% (3/5)
Gentamicin (10 μg)	2.0%	20% (1/5)
Imipenem (10 μg)	5.3%	0% (0/5)

**Fig 3:**
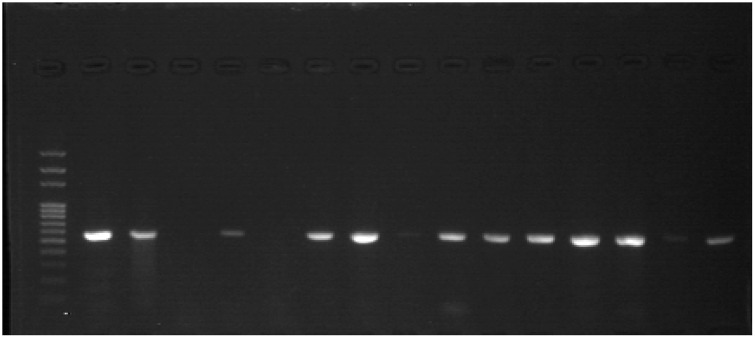
PCR amplification of the *bla*CTX-M gene in ESBL-producing *Salmonella enterica* isolates. Lane M contains a 100 bp DNA ladder. Lanes 1–15 show PCR products from representative isolates. The expected amplicon size of 552 bp confirms the presence of the *bla*CTX-M gene.

**Fig 4:**
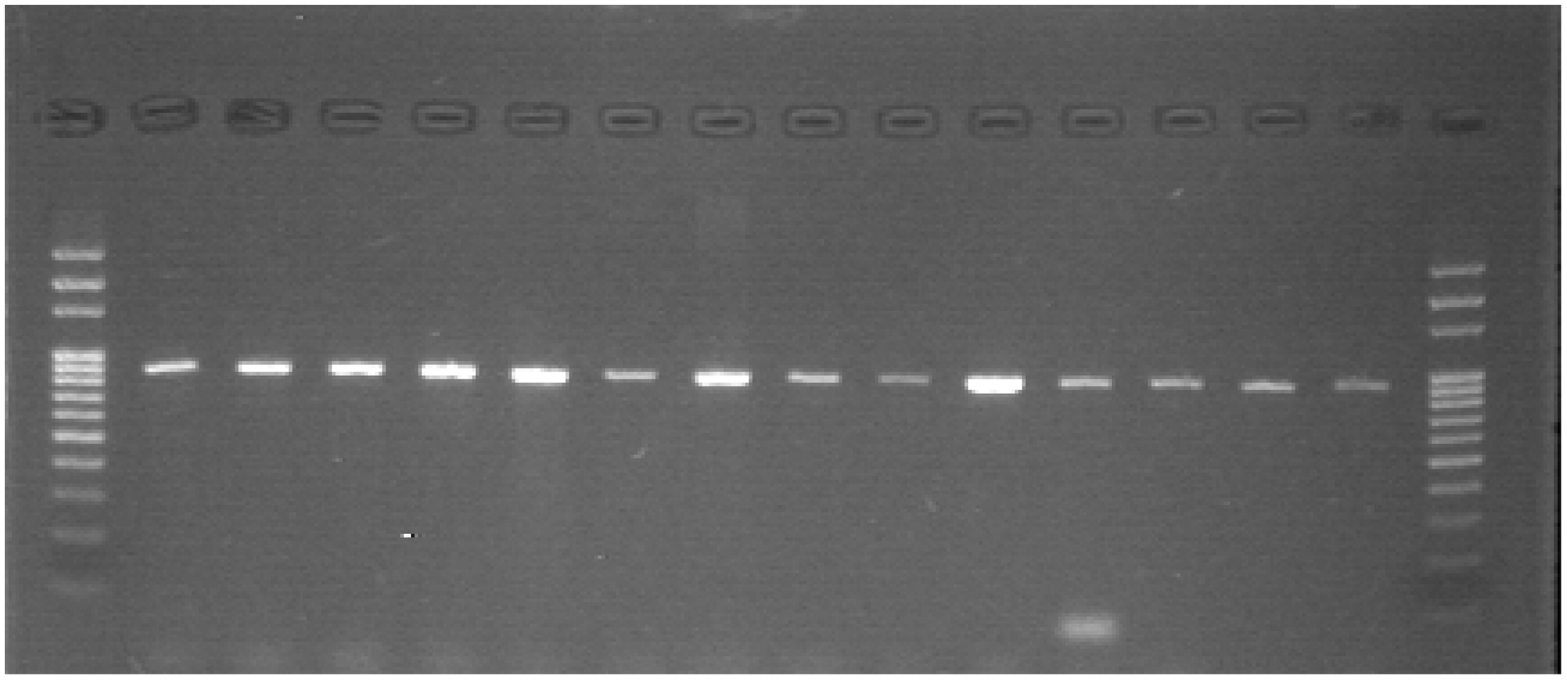
PCR amplification of the *bla*TEM gene in ESBL-producing *Salmonella enterica* isolates. Lane M contains a 100 bp DNA ladder. Lanes 1–15 show PCR products from representative isolates. The expected amplicon size of 856 bp confirms the presence of the *bla*TEM gene.

Genotypic characterization of the five ESBL-producing isolates was performed by PCR amplification of β-lactamase-encoding genes. The *bla*CTX-M gene (552 bp) was confirmed in three isolates (Fig 3), while the *bla*TEM gene (856 bp) was detected in three isolates (Fig 4). One isolate (isolate 68) was also found to harbour the *bla*SHV-12 gene.

The phenotypic and genotypic profiles of the five confirmed ESBL-producing isolates are summarized in Table 4. The five ESBL-positive *Salmonella* isolates (52, 53, 64, 66, 68) all displayed the core phenotypic profile of resistance to ampicillin, amoxicillin-clavulanate, ceftriaxone, and ceftazidime, but exhibited diverse multidrug-resistance patterns to agents including tetracycline, trimethoprim-sulfamethoxazole, ciprofloxacin, and gentamicin.

**Table 4:** Phenotypic and genotypic profiles of the ESBL-producing *Salmonella enterica* isolates (n = 5).

Isolate ID	Resistance profile	Associated resistance genes	Phylogenetic group (clone)
52	AMP, AMC, CRO, CAZ, TE, SXT	*bla*TEM	C18
53	AMP, AMC, CRO, CAZ, CIP, TE	*bla*CTX-M-15	C12
64	AMP, AMC, CRO, CAZ, CN, SXT	*bla*CTX-M-15, *bla*TEM	C12
66	AMP, AMC, CRO, CAZ, TE, CIP, SXT	*bla*TEM	C13
68	AMP, AMC, CRO, CAZ, TE, SXT, CIP	*bla*CTX-M-15, *bla*SHV-12	C46

Abbreviations: AMP, ampicillin; AMC, amoxicillin-clavulanate; CRO, ceftriaxone; CAZ, ceftazidime; TE, tetracycline; SXT, trimethoprim-sulfamethoxazole; CIP, ciprofloxacin; CN, gentamicin.

### Genetic diversity analysis by RAPD-PCR

**Fig 5:**
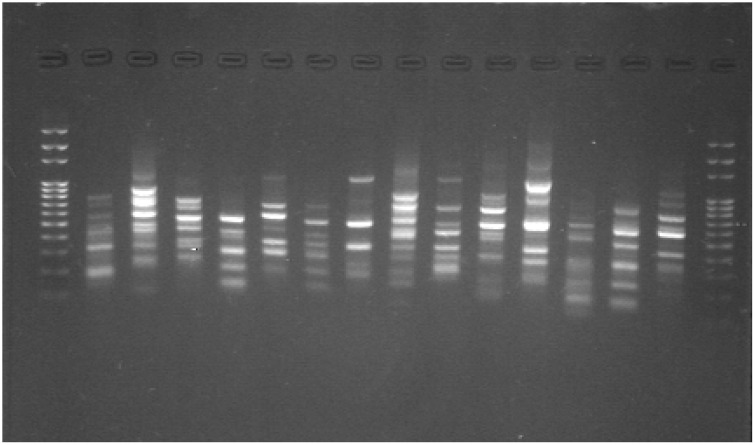
RAPD-PCR banding patterns of *Salmonella enterica* isolates generated using primer OPS-11. Lane M contains a 100 bp DNA ladder. Lanes 1–15 show representative RAPD profiles from 15 isolates. The polymorphic banding patterns illustrate the genetic diversity among isolates and served as the basis for phylogenetic analysis.

The genetic diversity of all 150 *Salmonella enterica* isolates was assessed using RAPD-PCR with primer OPS-11. The amplification generated reproducible and polymorphic banding patterns ranging from approximately 200 bp to 2000 bp. Representative RAPD profiles from 15 isolates are shown in Fig 5. The presence of distinct banding patterns across different isolates indicated substantial genetic heterogeneity within the sampled population. The binary scoring of these banding patterns served as the input data for phylogenetic reconstruction.

### Phylogenetic and phenotypic association analysis

**Fig 6:**
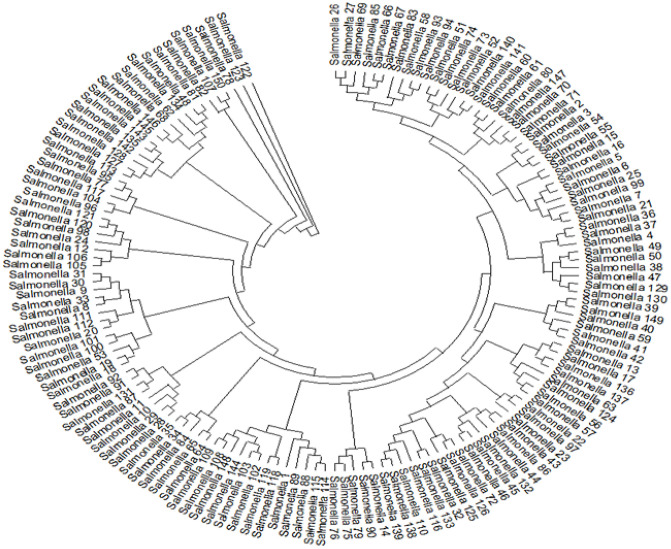
UPGMA dendrogram illustrating the phylogenetic relationships among 150 *Salmonella enterica* isolates recovered from retail beef samples in Peshawar, Pakistan. The dendrogram was constructed using MEGA 11 software based on RAPD-PCR banding patterns and the Nei and Li similarity coefficient. A similarity threshold of ≥ 75% was used to define clones, resulting in the identification of 58 distinct clones (C1–C58). The scale bar represents genetic distance. The distribution of isolates across multiple clusters indicates substantial genetic diversity, while the clustering of isolates from different geographic locations suggests possible cross-contamination during slaughtering, processing, or distribution.

The Nei and Li coefficient was used to generate a pairwise distance matrix from the RAPD banding data, with values ranging from 0 to 1. The distance matrix was used to construct a phylogenetic tree in MEGA 11, as shown in Fig 6.

A total of 58 clones (C1–C58) were identified based on a similarity coefficient threshold of ≥ 75%. Most clones contained two or three isolates, while four clones (C19, C27, C28, and one other) comprised four isolates each, suggesting limited clonal expansion of these specific genotypes. Several clones represented pairs (e.g., C4, C8, C13, C18), and a few contained unique isolates grouped with similar ones (e.g., C10, C17). Two isolates (isolates 1 and 16) were unique and did not cluster with any other isolate at the 75% similarity threshold. There was high genetic similarity between clones from the same geographic area, indicating potential clonal spread of *Salmonella* in the beef supply chain.

Phylogenetic clustering revealed that the five ESBL-positive isolates were not randomly distributed but were concentrated within three specific clones (C12, C13, and C46). Statistical analysis (Table 5) indicated significant genetic divergence between these ESBL-associated clones and the larger population (p < 0.05). Grubbs’ test identified no significant genetic outliers. The strong phylogenetic linkage, coupled with a trend toward the co-association of *bla*TEM and *bla*CTX-M genes, indicates a pattern of clonal spread.

**Table 5:** Statistical analysis of the association between phylogenetic clustering and ESBL/multidrug-resistance phenotypes.

Category	Comparison	Test applied	Result (p value / statistic)	Interpretation
Phylogenetic clustering	C12/C13/C46 vs. all other clones	Mann–Whitney U test	U = 15, p = 0.021	Significant genetic divergence (p < 0.05)
Outlier analysis	All ESBL+ isolates vs. population	Grubbs’ test (genetic distance)	G = 1.75, p = 0.402	No significant outliers
Resistance gene association	*bla*TEM vs. *bla*CTX-M distribution	Chi-square (χ²)	χ² = 3.20, p = 0.074	Trend toward association (not significant)
Resistance profile	CIP/TE resistance in C12/C13/C46	Fisher’s exact test	p = 0.035	Significant association (p < 0.05)
Clone–phylogeny link	ESBL+ phenotype in C12/C13/C46	Fisher’s exact test	p = 0.001	Highly significant linkage (p < 0.01)

## Discussion

This study employed RAPD-PCR and Nei and Li genetic diversity analysis to characterize *Salmonella* strains isolated from beef samples. The findings revealed a high prevalence (60.0%) of *Salmonella enterica*, substantial genetic diversity, and widespread multidrug resistance (MDR). Phenotypic and *invA*-based molecular identification confirmed *Salmonella enterica*, whereas RAPD-PCR clustered the isolates into distinct phylogenetic groups. Some clusters were associated with specific antimicrobial resistance patterns, including ESBL-producing strains harbouring *bla*TEM, *bla*CTX-M, and *bla*SHV genes. The findings highlight beef as a reservoir for diverse and resistant *Salmonella* strains. This underscores the need for enhanced surveillance in food safety and public health interventions.

The high isolation rate (60.0%) of *Salmonella enterica* from beef samples in this study aligns with previous reports from developing regions. In such settings, poor hygiene and inadequate cold-chain maintenance exacerbate contamination [17,18]. However, this prevalence exceeds rates reported in high-income countries; for example, rates of 5–20% are common in the European Union and the United States. This difference is likely due to stricter regulatory enforcement and advanced slaughterhouse practices in those regions [19]. The reliance on conventional culture methods (XLD/SS agar and biochemical tests) mirrors ISO 6579–1:2017 protocols, which remain the gold standard for *Salmonella* detection [20]. Nevertheless, these methods have limitations. In this study, a small proportion of isolates presented atypical H_2_S-negative phenotypes on TSI agar. Such atypical reactions could potentially lead to false negatives if used alone [8]. Similar discrepancies were noted by Gad et al., who reported that 4% of *Salmonella* strains from poultry deviated from standard biochemical profiles [21]. While phenotypic tests are cost-effective, their specificity is limited by closely related Enterobacteriaceae; for instance, *Citrobacter* spp. can produce similar colonial morphology [22]. Molecular confirmation via *invA* PCR mitigated this issue in the present study, supporting the findings of Malorny et al., who reported 100% specificity for *invA* in *Salmonella* differentiation [23]. In contrast, some studies argue that *invA* may miss rare serovars lacking this gene [24], suggesting that supplemental markers such as *ttr* could enhance detection sensitivity. The higher prevalence observed in rural samples (77.5%) echoes findings from Ethiopia [25] and India [26], linking contamination to unregulated slaughter practices and limited infrastructure. Conversely, urban samples presented lower rates (52.5%), possibly due to better infrastructure; however, these urban rates remain higher than those reported in Brazil (20–30%) [27]. These disparities underscore the need for context-specific interventions, as emphasized by WHO guidelines for low-resource settings.

The use of *invA* PCR for molecular confirmation in this study aligns with established protocols [28] and reinforces the reliability of *invA* as a species-specific marker. Malorny et al. demonstrated 100% specificity across 111 *Salmonella* serovars [23]. This high specificity is critical in beef matrices, where background microbiota such as *E. coli* and *Citrobacter* can mimic phenotypic traits of *Salmonella* [29]. In this study, detection of *invA* in all phenotypically confirmed isolates (100%) supports its utility in resource-limited settings. This approach is advocated by ISO 6579–1:2017 for unambiguous identification. Nevertheless, dependence on the exclusive use of *invA* has limitations. Although it is highly conserved, rare serovars such as *S. enterica* subsp. *arizonae* may lack this gene, and false negatives may consequently occur [24]. This limitation contrasts with studies that promote multigene approaches (e.g., *ttr* and *hilA*) for broader coverage [30]. For example, Park et al. reported that *hilA* is a complementary target with 99.5% sensitivity in clinical *Salmonella* isolates, including *invA*-negative strains [31]. The findings of this study also inform the discussion of PCR sensitivity in complex food matrices. Although *invA* PCR performed well here, other reports have noted inhibitory effects of beef-derived substances such as fats and collagen, which often require optimization of DNA extraction protocols [32]. In the present study, the use of commercial DNA extraction kits likely addressed these concerns, consistent with the recommendations of Seethalakshmi et al., who emphasized kit-based extraction for stable PCR performance in faecal and food samples [33].

The application of RAPD-PCR in this study successfully revealed high genetic variability among *Salmonella enterica* isolates from beef, demonstrated by the division of isolates into 58 distinct phylogenetic clusters [34]. This approach is comparable to previous studies that employed RAPD-PCR to determine the population structure of *Salmonella* [35,36]. The ability of this technique to distinguish closely related strains without prior genomic knowledge makes it valuable for outbreak tracing and source attribution in foodborne pathogens [37]. However, the reproducibility of RAPD-PCR has been questioned, as the technique is sensitive to experimental conditions, including primer concentrations and PCR cycling parameters [38]. In the present study, phylogenetic analysis yielded 58 distinct clones (C1–C58) based on a similarity threshold of ≥ 75%. Most clones contained two or three isolates, and four clones (C19, C27, C28, and one other) comprised four isolates each. This pattern suggests limited clonal expansion of these specific genotypes. Two isolates (isolates 1 and 16) were unique and did not cluster with any other isolate. This level of diversity is consistent with studies on poultry-derived *Salmonella* as measured by the Nei and Li coefficient and Simpson index [39]. However, contrasting results have been reported in other settings, and such differences can be attributed to variation in selective pressures between beef and poultry production systems [40].

Notably, the clustering pattern revealed that genetically similar isolates were not confined to a single geographical region. This suggests possible cross-contamination events during slaughtering, processing, or distribution stages. Several clusters contained isolates from both urban and rural sampling sites, supporting the hypothesis of overlapping contamination sources. The presence of closely related strains across different market types also implies the persistence or reintroduction of dominant genotypes over time. This pattern has been described previously by Liao et al., who documented cross-contamination risk in food-contact environments [41].

A key finding of this study was the observation of ESBL-producing strains in distinct phylogenetic clusters. The five ESBL-positive isolates were concentrated within three specific clones: C12, C13, and C46. Statistical analysis confirmed significant genetic divergence between these ESBL-associated clones and the larger population (p < 0.05). This finding supports clonal expansion as a primary dissemination mechanism rather than random acquisition of resistance. The co-occurrence of *bla*TEM and *bla*CTX-M genes in isolate 64, and the presence of *bla*SHV-12 in isolate 68, further underscore the accumulation of multiple resistance determinants within successful clonal lineages. This pattern of clonal dissemination of ESBL-producing *Salmonella* is consistent with reports by Ibrahim et al., who documented similar trends in clinical isolates [42].

While RAPD-PCR offers clear benefits, it has lower resolution compared to contemporary methods such as whole-genome sequencing (WGS) [43]. WGS provides comprehensive genomic information, including single nucleotide polymorphisms and detailed resistome analysis. However, in resource-constrained contexts, RAPD-PCR remains a cost-friendly and accessible option for initial screening and epidemiological surveillance [44]. The use of a single primer (OPS-11) in this study is standard practice and has been widely employed for *Salmonella* typing [12]. Nevertheless, some researchers advocate for multiple-primer strategies to increase discriminatory power and improve reproducibility. This limitation is acknowledged and should be addressed in future investigations.

The use of the Nei and Li coefficient in this analysis complemented the RAPD-PCR dataset effectively. This coefficient allowed an objective comparison of banding patterns and the calculation of pairwise genetic distances, an approach that aligns with the original work of Nei and Li, who demonstrated its utility in measuring genetic variation [45]. It has also been applied successfully in subsequent bacterial phylogenetic studies [36]. The identification of distinct phylogenetic clusters with varying genetic distances supports the technique’s sensitivity in differentiating closely related strains in foodborne *Salmonella* outbreaks [46]. However, reliance on shared band presence/absence data has limitations compared to sequence-based methods, as this approach cannot account for nucleotide-level variations that might influence evolutionary relationships [47]. The detection of dominant lineages with this approach is consistent with previously reported clonal spread of beef-associated *Salmonella* [48]. Nonetheless, the genetic heterogeneity observed here, particularly among ESBL-producing isolates, appears less homogeneous than that reported in poultry-derived *Salmonella* [49], suggesting that different selective pressures may operate across livestock industries.

## Conclusion

This study characterized *Salmonella enterica* isolates recovered from retail beef samples in Peshawar, Pakistan, using a combined phenotypic and molecular approach. The overall prevalence of *S. enterica* was 60.0% (150/250), with the highest contamination observed in samples from rural markets (77.5%). Antimicrobial susceptibility testing revealed that a high percentage of isolates were resistant to azithromycin (94.0%), tetracycline (68.7%), and streptomycin (52.0%), indicating widespread multidrug resistance. Five isolates (3.3%) were confirmed as ESBL producers and harboured *bla*TEM-1, *bla*CTX-M-15, and *bla*SHV-12 resistance genes. RAPD-PCR analysis revealed substantial genetic diversity, with isolates distributed across 58 distinct phylogenetic clusters. The ESBL-producing isolates were not randomly distributed but were concentrated within three specific clones, suggesting clonal dissemination of resistant strains. These findings demonstrate that retail beef in Peshawar serves as a significant reservoir of genetically diverse and antimicrobial-resistant *S. enterica*. The integrated use of phenotypic methods with targeted molecular and low-cost genotyping approaches provides a practical surveillance framework for foodborne pathogens in resource-limited settings.

This study has several limitations. Phenotypic identification methods lack discriminatory power for closely related strains and may yield false-negative results for atypical isolates. Although highly specific, *invA* PCR may fail to detect rare serovars lacking this gene. The RAPD-PCR method, while cost-effective, is sensitive to experimental variation and offers lower resolution than whole-genome sequencing. Additionally, the use of a single RAPD primer may have limited the discriminatory power of the genetic diversity analysis. The geographic scope was limited to a single district, which may affect the generalizability of the findings. Finally, the analysis of ESBL-producing isolates was based on a small subset (n = 5). Future studies should employ multigene molecular assays alongside *invA* PCR to improve detection accuracy, apply whole-genome sequencing for higher-resolution phylogenetic analysis and source tracking, and use multiple RAPD primers with standardized protocols to enhance reproducibility and discriminatory power. Expanding the geographic scope and sample size, together with longitudinal surveillance, would provide a more comprehensive understanding of resistance trends and strain dynamics over time. Region-specific monitoring programmes and improved sanitary measures at slaughterhouses and retail markets are urgently needed to mitigate the public health risks posed by multidrug-resistant and ESBL-producing *Salmonella* in the food supply chain.

## Supporting information

S1 File The supporting information included in this manuscript comprised of the original uncropped images. (DOCX)

S1 Raw Image Raw image. (PDF)
