# Supplementary material for: Phenotypic and molecular characterization of Salmonella enterica isolated from retail beef in Peshawar, Pakistan
Source: PLoS One. 2026 Jul 29;21(7):e0352859. doi: 10.1371/journal.pone.0352859 (PMC13419216; doi:10.1371/journal.pone.0352859)
Supplement: S1 File — (DOCX) [file pone.0352859.s001.docx]

**S1_Data**

**Gel image compilation and annotations**

*Phenotypic and Molecular Characterization of Salmonella enterica Isolated from Retail Beef in Peshawar, Pakistan*

**Included gel image annotations**

| **Item** | **Related panel** | **Gel / assay** | **Marker / lanes** |
| --- | --- | --- | --- |
| **Fig 1** | Manuscript Figure 1 | Agarose gel electrophoresis of extracted genomic DNA from representative Salmonella enterica isolates | 100 bp DNA ladder; lanes 1-15 |
| **Fig 2** | Manuscript Figure 2 | PCR amplification of the invA gene for species-level confirmation of Salmonella enterica | 100 bp DNA ladder; lanes 1-15 |
| **Fig 3** | Manuscript Figure 3 | PCR amplification of the blaCTX-M gene in ESBL-producing Salmonella enterica isolates | 100 bp DNA ladder; lanes 1-15 |
| **Fig 4** | Manuscript Figure 4 | PCR amplification of the blaTEM gene in ESBL-producing Salmonella enterica isolates | 100 bp DNA ladder; lanes 1-15 |
| **Fig 5** | Manuscript Figure 5 | RAPD-PCR banding patterns of Salmonella enterica isolates generated using primer OPS-11 | 100 bp DNA ladder; lanes 1-15 |

**Fig 1. Agarose gel electrophoresis of extracted genomic DNA from representative Salmonella enterica isolates**


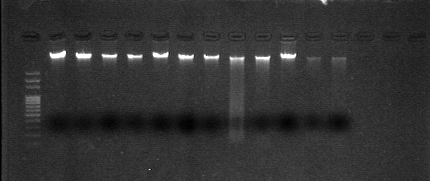


| **Related panel** | Manuscript Figure 1 |
| --- | --- |
| **Loading order / lanes** | Lane M: 100 bp DNA ladder; lanes 1-15: genomic DNA from representative Salmonella enterica isolates. |
| **Sample identity** | Representative Salmonella enterica isolates, lanes 1-15. |
| **Capture / visualization method** | Genomic DNA agarose gel electrophoresis; UV visualization. |
| **Molecular-weight marker** | 100 bp DNA ladder. |
| **Excluded lanes** | No lanes marked as excluded. |

**Fig 2. PCR amplification of the invA gene for species-level confirmation of Salmonella enterica**


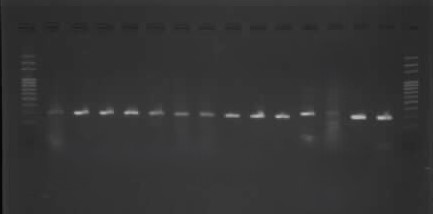


| **Related panel** | Manuscript Figure 2 |
| --- | --- |
| **Loading order / lanes** | Lane M: 100 bp DNA ladder; lanes 1-15: PCR products from representative isolates; expected amplicon size 284 bp. |
| **Sample identity** | Representative Salmonella enterica isolates screened by invA PCR, lanes 1-15. |
| **Capture / visualization method** | PCR products separated by agarose gel electrophoresis; UV visualization. |
| **Molecular-weight marker** | 100 bp DNA ladder. |
| **Excluded lanes** | No lanes marked as excluded. |

**Fig 3. PCR amplification of the blaCTX-M gene in ESBL-producing Salmonella enterica isolates**


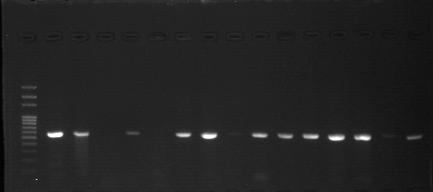


| **Related panel** | Manuscript Figure 3 |
| --- | --- |
| **Loading order / lanes** | Lane M: 100 bp DNA ladder; lanes 1-15: PCR products from representative isolates; expected amplicon size 552 bp. |
| **Sample identity** | Salmonella enterica isolates screened for blaCTX-M, lanes 1-15. |
| **Capture / visualization method** | PCR products separated by agarose gel electrophoresis; UV visualization. |
| **Molecular-weight marker** | 100 bp DNA ladder. |
| **Excluded lanes** | No lanes marked as excluded. |

**Fig 4. PCR amplification of the blaTEM gene in ESBL-producing Salmonella enterica isolates**


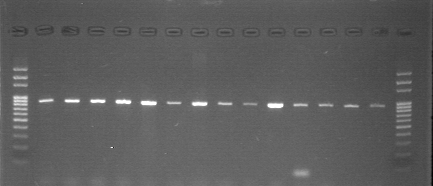


| **Related panel** | Manuscript Figure 4 |
| --- | --- |
| **Loading order / lanes** | Lane M: 100 bp DNA ladder; lanes 1-15: PCR products from representative isolates; expected amplicon size 856 bp. |
| **Sample identity** | Salmonella enterica isolates screened for blaTEM, lanes 1-15. |
| **Capture / visualization method** | PCR products separated by agarose gel electrophoresis; UV visualization. |
| **Molecular-weight marker** | 100 bp DNA ladder. |
| **Excluded lanes** | No lanes marked as excluded. |

**Fig 5. RAPD-PCR banding patterns of Salmonella enterica isolates generated using primer OPS-11**


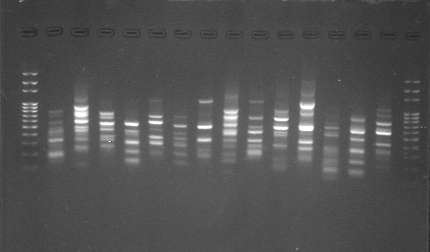


| **Related panel** | Manuscript Figure 5 |
| --- | --- |
| **Loading order / lanes** | Lane M: 100 bp DNA ladder; lanes 1-15: representative RAPD profiles; observed banding range approximately 200-2000 bp. |
| **Sample identity** | Representative Salmonella enterica isolates used for RAPD-PCR genetic diversity analysis, lanes 1-15. |
| **Capture / visualization method** | RAPD-PCR products separated by agarose gel electrophoresis; UV visualization. |
| **Molecular-weight marker** | 100 bp DNA ladder. |
| **Excluded lanes** | No lanes marked as excluded. |


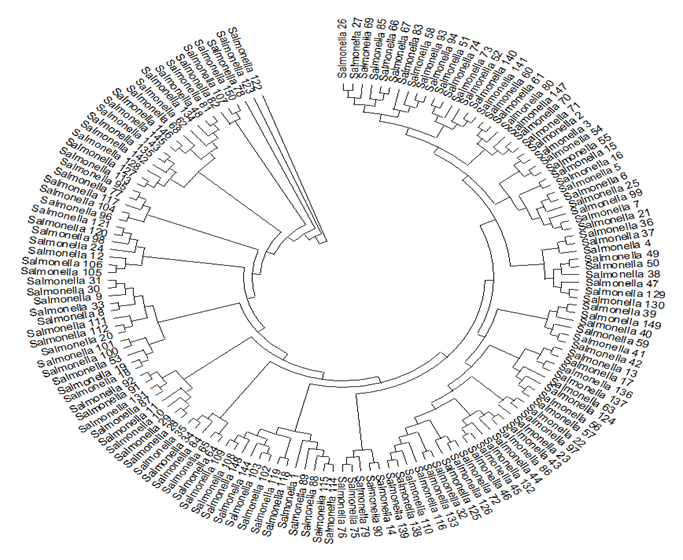


**Fig 6. UPGMA dendrogram illustrating the phylogenetic relationships among 150** *Salmonella enterica* isolates recovered from retail beef samples in Peshawar, Pakistan. The dendrogram was constructed using MEGA 11 software based on RAPD-PCR banding patterns and the Nei and Li similarity coefficient. A similarity threshold of ≥ 75% was used to define clones, resulting in the identification of 58 distinct clones (C1–C58). The scale bar represents genetic distance. The distribution of isolates across multiple clusters indicates substantial genetic diversity, while the clustering of isolates from different geographic locations suggests possible cross-contamination during slaughtering, processing, or distribution.
