# Supplementary material for: Phenotypic and molecular characterization of Salmonella enterica isolated from retail beef in Peshawar, Pakistan
Source: PLoS One. 2026 Jul 29;21(7):e0352859. doi: 10.1371/journal.pone.0352859 (PMC13419216; doi:10.1371/journal.pone.0352859)
Supplement: S1 Raw Image — (PDF) [file pone.0352859.s002.pdf]

S1_raw_images

Phenotypic and Molecular Characterization of Salmonella enterica Isolated from Retail Beef in Peshawar, Pakistan

This file contains the original, uncropped, and unadjusted agarose-gel images underlying all gel data reported in the manuscript and Supporting Information. Five gel images are provided, corresponding to Manuscript Figures 1–5. Figure 6 (UPGMA dendrogram) is a computational output, not a gel/blot, and is therefore not included here. Each image is shown at full resolution without down-sampling. All loading-order, lane, and marker annotations are placed in the page margins so that no gel data or background bands are obscured. Molecular-weight markers (100 bp DNA ladder) are indicated on every image. No lanes were excluded from the final figures; therefore no lanes are marked with “X”.

Index of raw images

Item	Related panel	Gel / assay
Fig 1	Manuscript Figure 1	Agarose gel electrophoresis of extracted genomic DNA from representative Salmonella enterica isolates
Fig 2	Manuscript Figure 2	PCR amplification of the invA gene for species-level confirmation of Salmonella enterica
Fig 3	Manuscript Figure 3	PCR amplification of the blaCTX-M gene in ESBL-producing Salmonella enterica isolates
Fig 4	Manuscript Figure 4	PCR amplification of the blaTEM gene in ESBL-producing Salmonella enterica isolates
Fig 5	Manuscript Figure 5	RAPD-PCR banding patterns of Salmonella enterica isolates generated using primer OPS-11

Fig 1 – raw gel image

Agarose gel electrophoresis of extracted genomic DNA from representative Salmonella enterica isolates

M 1 2 3 4 5 6 7 8 9 10 11 12 13 14 15

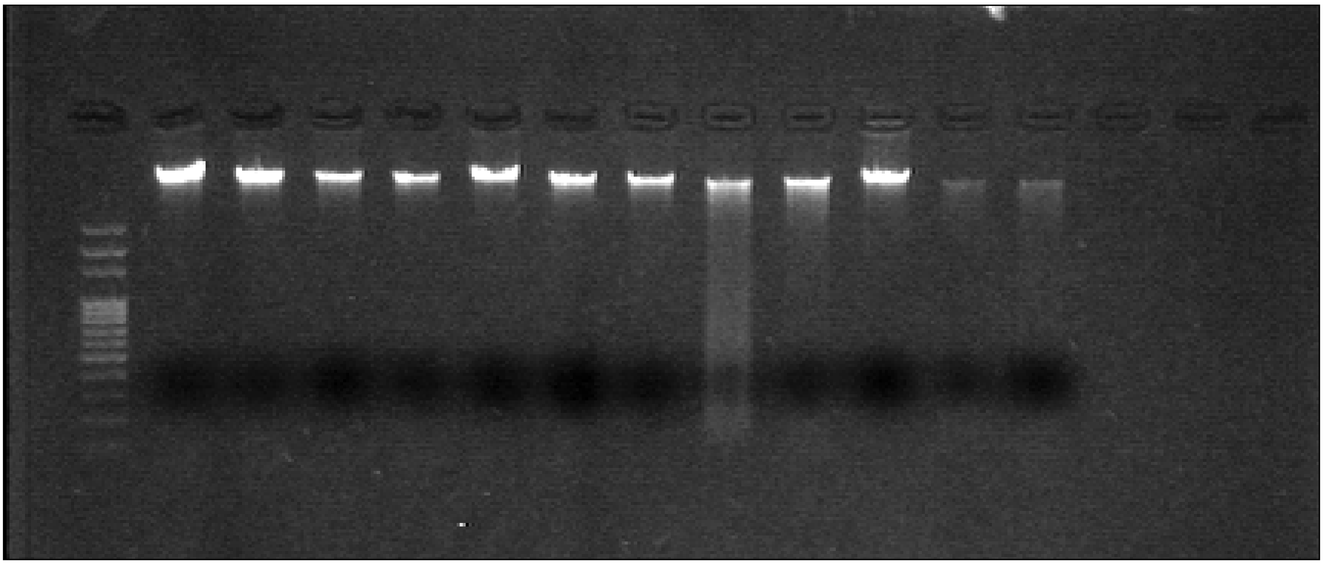

Lane labels above the image indicate loading order (M = molecular-weight marker lane). Labels are spaced across the lane region and sit in the margin above the wells; they do not overlie any band or background signal.

**Related panel:**	Manuscript Figure 1
**Loading order / lanes:**	Lane M: 100 bp DNA ladder (left). Lanes 1–15: genomic DNA from 15 representative S. enterica isolates.
**Identity of experimental samples:**	Genomic DNA of 15 representative S. enterica isolates (lanes 1–15).
**Method used to capture the image:**	Genomic DNA separated on 1.5% agarose gel; ethidium-bromide staining; UV transillumination / gel-documentation capture.
**Molecular-weight marker:**	100 bp DNA ladder (lane M, left).
**Lanes excluded from final figure:**	None. All lanes shown in this image were used in Manuscript Figure 1.

Fig 2 – raw gel image

PCR amplification of the invA gene for species-level confirmation of Salmonella enterica (expected amplicon 284 bp)

M 1 2 3 4 5 6 7 8 9 10 11 12 13 14 15 M

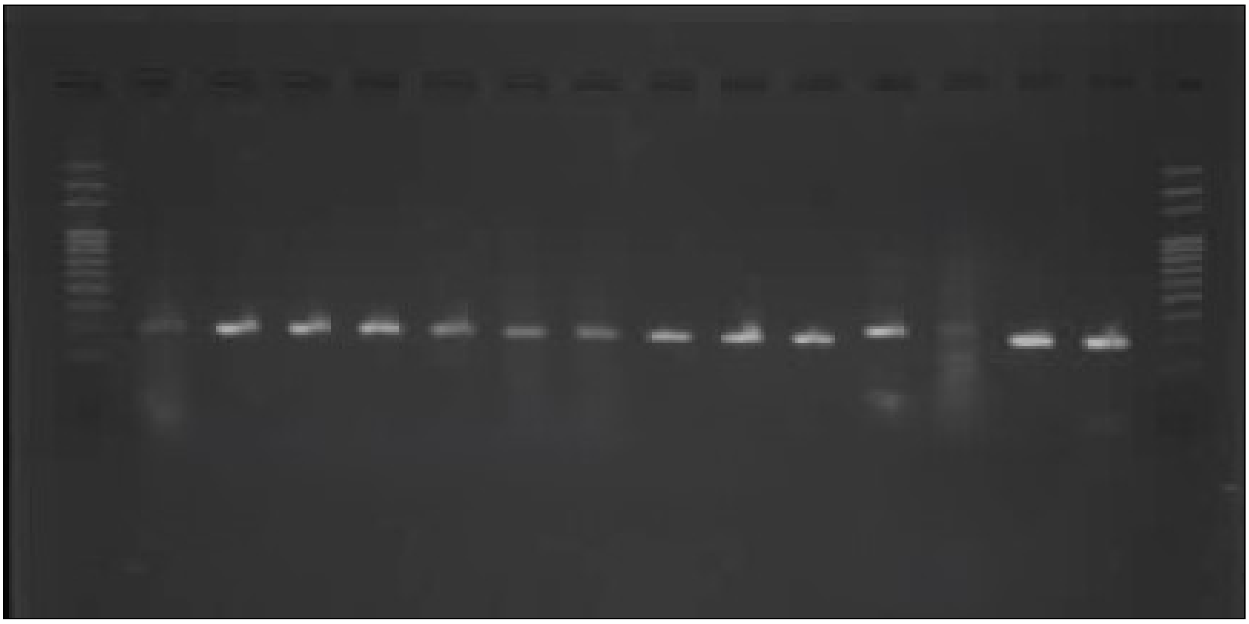

Lane labels above the image indicate loading order (M = molecular-weight marker lane). Labels are spaced across the lane region and sit in the margin above the wells; they do not overlie any band or background signal.

**Related panel:**	Manuscript Figure 2
**Loading order / lanes:**	Lane M: 100 bp DNA ladder (both outer lanes). Lanes 1–15: invA PCR products from representative isolates.
**Identity of experimental samples:**	Representative S. enterica isolates screened by invA PCR (lanes 1–15).
**Method used to capture the image:**	PCR products separated on 1.5% agarose gel; ethidium-bromide staining; UV transillumination / gel-documentation capture.
**Molecular-weight marker:**	100 bp DNA ladder (lanes M, left and right).
**Lanes excluded from final figure:**	None. All sample lanes shown were used in Manuscript Figure 2.

Fig 3 – raw gel image

PCR amplification of the blaCTX-M gene in ESBL-producing Salmonella enterica isolates (expected amplicon 552 bp)

M 1 2 3 4 5 6 7 8 9 10 11 12 13 14 15

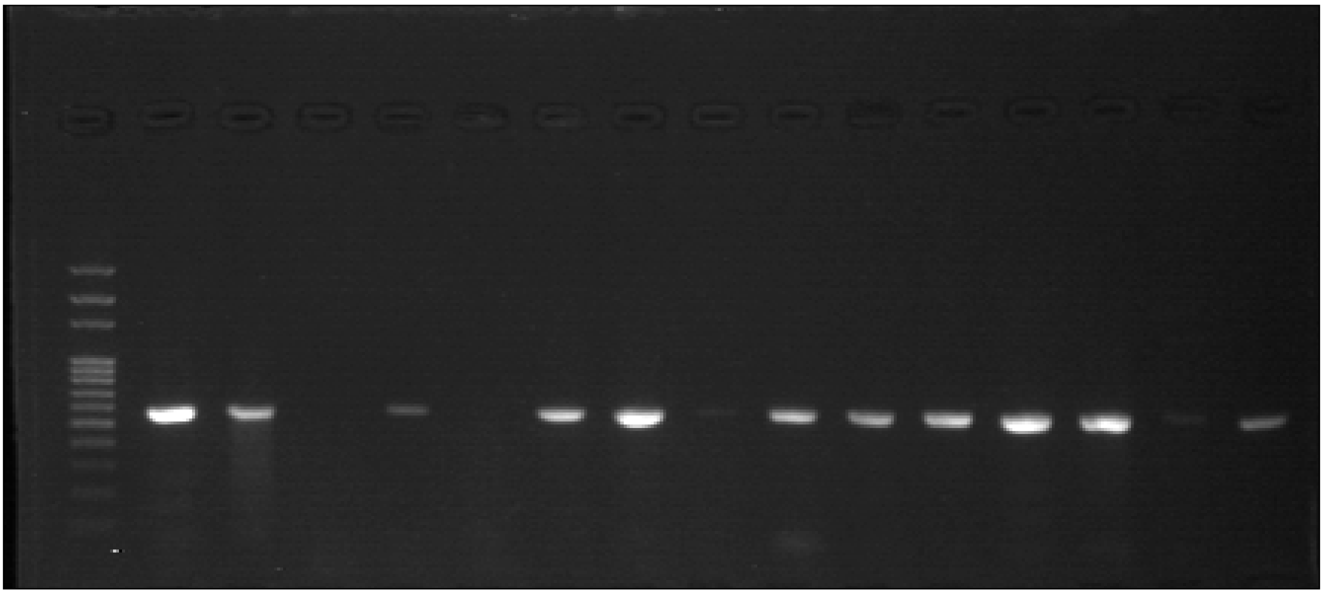

Lane labels above the image indicate loading order (M = molecular-weight marker lane). Labels are spaced across the lane region and sit in the margin above the wells; they do not overlie any band or background signal.

**Related panel:**	Manuscript Figure 3
**Loading order / lanes:**	Lane M: 100 bp DNA ladder (left). Lanes 1–15: blaCTX-M PCR products from representative isolates.
**Identity of experimental samples:**	S. enterica isolates screened for blaCTX-M (lanes 1–15).
**Method used to capture the image:**	PCR products separated on 1.5% agarose gel; ethidium-bromide staining; UV transillumination / gel-documentation capture.
**Molecular-weight marker:**	100 bp DNA ladder (lane M, left).
**Lanes excluded from final figure:**	None. All lanes shown were used in Manuscript Figure 3.

Fig 4 – raw gel image

PCR amplification of the blaTEM gene in ESBL-producing Salmonella enterica isolates (expected amplicon 856 bp)

M 1 2 3 4 5 6 7 8 9 10 11 12 13 14 15 M

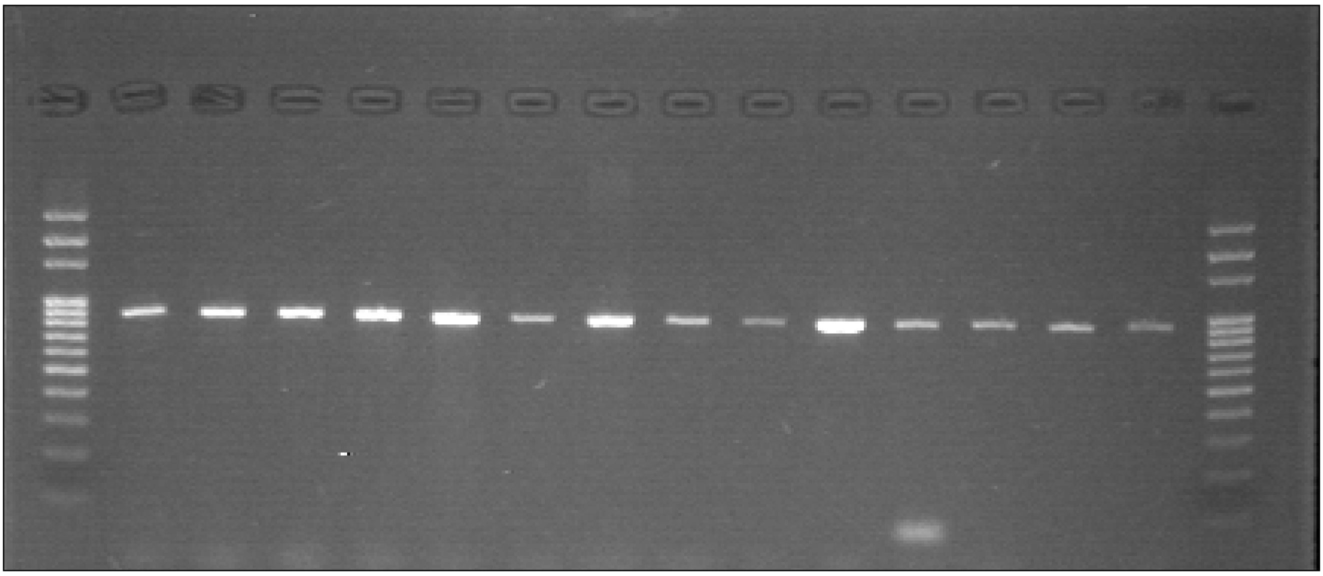

Lane labels above the image indicate loading order (M = molecular-weight marker lane). Labels are spaced across the lane region and sit in the margin above the wells; they do not overlie any band or background signal.

**Related panel:**	Manuscript Figure 4
**Loading order / lanes:**	Lane M: 100 bp DNA ladder (both outer lanes). Lanes 1–15: blaTEM PCR products from representative isolates.
**Identity of experimental samples:**	S. enterica isolates screened for blaTEM (lanes 1–15).
**Method used to capture the image:**	PCR products separated on 1.5% agarose gel; ethidium-bromide staining; UV transillumination / gel-documentation capture.
**Molecular-weight marker:**	100 bp DNA ladder (lanes M, left and right).
**Lanes excluded from final figure:**	None. All sample lanes shown were used in Manuscript Figure 4.

Fig 5 – raw gel image

RAPD-PCR banding patterns of Salmonella enterica isolates generated using primer OPS-11 (banding range ~200–2000 bp)

M 1 2 3 4 5 6 7 8 9 10 11 12 13 14 15 M

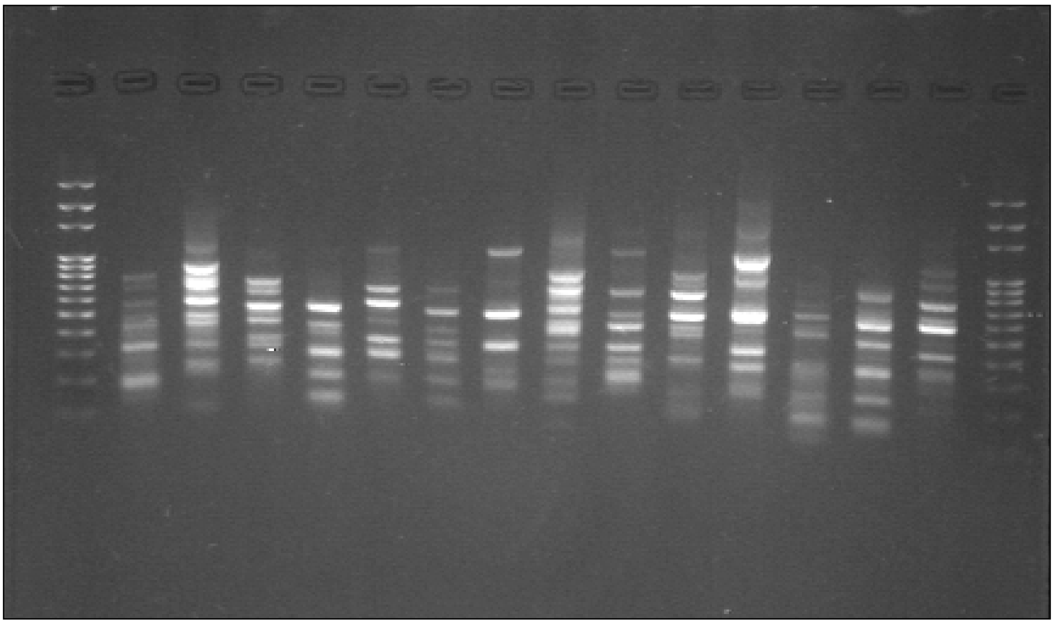

Lane labels above the image indicate loading order (M = molecular-weight marker lane). Labels are spaced across the lane region and sit in the margin above the wells; they do not overlie any band or background signal.

**Related panel:**	Manuscript Figure 5
**Loading order / lanes:**	Lane M: 100 bp DNA ladder (both outer lanes). Lanes 1–15: representative RAPD-PCR profiles.
**Identity of experimental samples:**	Representative S. enterica isolates used for RAPD-PCR genetic diversity analysis (lanes 1–15).
**Method used to capture the image:**	RAPD-PCR products separated on 1.5% agarose gel; ethidium-bromide staining; UV transillumination / gel-documentation capture.
**Molecular-weight marker:**	100 bp DNA ladder (lanes M, left and right).
**Lanes excluded from final figure:**	None. All sample lanes shown were used in Manuscript Figure 5.
