# Supplementary material for: Phenotypic and molecular characterization of Salmonella enterica isolated from retail beef in Peshawar, Pakistan
Source: PLoS One. 2026 Jul 29;21(7):e0352859. doi: 10.1371/journal.pone.0352859 (PMC13419216; doi:10.1371/journal.pone.0352859)
Supplement: Attachment — Submitted filename: PONE-D-26-0-1381.docx [file pone.0352859.s003.docx]

Title of the manuscript: Phenotypic and molecular diversity analysis of *Salmonella enterica* contaminating beef samples sold in Peshawar, Pakistan

The authors described the diversity of *Salmonella enterica* from beef samples sold in Peshawar, Pakistan. This manuscript is of interest for limited readerships. It has many places where improvement can be done. I recommend for major revision of the manuscript.

1. Abstract, the names of genes should be written as *blaTEM*, *blaCTX-M* (italic). Why did you not explore *blaSHV* among ESBL producer?

2. Please make the name of bacteria italic throughout the manuscript.

3. Methods, please provide the reference for ESBL screening and confirmatory test.

4. If you use the primers from already published article, please provide the reference.

5. Results, please revise the sub-title, “demographic distribution of *Salmonella enterica* isolates”. You can just say “Distribution of *Salmonella enterica* isolates”.

6. The sub-title “Culturing and antimicrobial sensitivity analysis” should be revised. You can write “Antimicrobial susceptibility of the *Salmonella enterica* isolates”.

7. The text description of Table 2 should be revised. “High resistance was observed to azithromycin (94.0%), tetracycline (68.7%), and streptomycin (52.0%), indicating widespread multidrug resistance”. In fact, it is not high resistance. It means “High percentage of isolates were resistant to azithromycin (94.0%), tetracycline (68.7%), and streptomycin (52.0%).” Similarly, antibiotics are not resistant. Bacteria is resistant to antibiotics. Please revise other sentences in the same paragraph.

8. Please do not keep discussion sentences in the results.

9. In results of abstract, it is mentioned that “Five isolates (3.3%) were confirmed as extended-spectrum ß-lactamase (ESBL) producers…………..” but in results in page 14, it has been mentioned as “Among the 150 Salmonella enterica isolates, four (2.7%) were confirmed as extended-spectrum ß-lactamase (ESBL) producers …………..”. In following sentences, there is also five ESBL producers.

10. Please revise the title of Table 3 “ESBL-producing isolates (n=5) exhibited significantly higher resistance rates compared to the overall population (n=150).” Further, you have not used p-value to test the significant association. Therefore, please delete “significantly”.

11. Table 3, column 2, better to write “Resistance in total isolates”.

12. Please provide the text description of Table 4, just before the table.

13. In methods, it has been mentioned that *blaCTX-M* gene specific primers were used. But in Table 4, it has given *blaCTX-M-15*. How did you detect *blaCTX-M-15*?

14. Table 5 and its text, please revise the sub-title and title of the Table 5. Please provide specific sub-title and title of the Table 5. It cannot be statistical analysis.

15. The text description of Page 18 is related to discussion. Please shift this paragraph to discussion.

16. Discussion, please make the name of genes italic.

17. “The results of this study, Showed that there were 58 clones, out of which 4 clones have maximum isolates of four and mostly were grouped in two or three isolates.” Not clear. Please revise.

18. There are many grammatical errors in the manuscript. Please correct those errors.

19. Conclusion should be brief, concise and based on results. Please keep only essentials in the conclusion.
