# Supplementary material for: Phenotypic and molecular characterization of Salmonella enterica isolated from retail beef in Peshawar, Pakistan
Source: PLoS One. 2026 Jul 29;21(7):e0352859. doi: 10.1371/journal.pone.0352859 (PMC13419216; doi:10.1371/journal.pone.0352859)
Supplement: Attachment — Submitted filename: Response_to_Reviewers.docx [file pone.0352859.s005.docx]

**Date:** 31 May 2026

**To:**

Dr. Dwij Raj Bhatta

Academic Editor, PLOS ONE

**Re:** Response to Reviewers – Manuscript PONE-D-26-01381 (Revised)

**Title:** Phenotypic and Molecular Characterization of *Salmonella enterica* Isolated from Retail Beef in Peshawar, Pakistan

Dear Dr. Bhatta,

We sincerely thank you and the reviewers for the time and effort invested in evaluating our manuscript. The comments were constructive and have substantially improved the clarity, rigour, and presentation of our work. We have carefully revised the manuscript to address every point raised by the editorial office and both reviewers.

Below we provide a detailed, point-by-point response to each comment. For every revision, we indicate the **page number** and **line number** in the revised manuscript file (“Revised_Manuscript_Highlighted.docx”), in which all changes are marked with **yellow highlighting** to facilitate verification. Line numbers refer to the continuous line numbering applied throughout the revised manuscript. A clean (unhighlighted) version is also provided. This response is organized into three parts:

• **Part A –** Response to Journal Requirements (Academic Editor)

• **Part B –** Response to Reviewer 1

• **Part C –** Response to Reviewer 2

We trust that the revisions fully address all concerns and that the manuscript now meets the publication standards of PLOS ONE. The page and line references below correspond to the revised manuscript file as submitted.

**Part A – Response to Journal Requirements (Academic Editor)**

| **#** | **Reviewer / Editor comment** | **Author response** | **Location in revised manuscript** |
| --- | --- | --- | --- |
| **A1** | Please ensure that your manuscript meets PLOS ONE’s style requirements, including those for file naming. | The manuscript has been fully reformatted to PLOS ONE style: Times New Roman 12 pt, double line spacing, continuous line numbering, page numbering, justified body text, PLOS-style figure labels (“Fig 1”–“Fig 6”) and table labels, and Vancouver-style references. Files are named according to journal conventions (Revised_Manuscript, Revised_Manuscript_Highlighted, Response_to_Reviewers). | **Entire manuscript** (formatting applied throughout). |
| **A2** | PLOS ONE requires the original uncropped and unadjusted images underlying all blot or gel results. | Original, uncropped, and unadjusted gel images for Figs 1–5 have been prepared and will be uploaded as Supporting Information (**S1 Raw Images**) at resubmission. All figures comply with the journal’s guidance on cropping, contrast adjustment, and labelling, as confirmed in the cover letter. | Noted in cover letter; **S1 Raw Images** uploaded separately. |
| **A3** | Please confirm that your Competing Interests statement does not alter your adherence to PLOS ONE policies on sharing data and materials. | The Competing Interests statement now reads: “The authors have declared that no competing interests exist. This does not alter our adherence to PLOS ONE policies on sharing data and materials.” | **Declarations – Competing interests** (Page 24). |
| **A4** | All data underlying the findings must be freely available without restriction. | All underlying datasets have been deposited in the Zenodo public repository and are freely available. The Data Availability Statement now includes the DOI link (https://zenodo.org/records/18700727; DOI: 10.5281/zenodo.18700727). | **Declarations – Availability of data and materials** (Page 24). |
| **A5** | Please amend the submission data to include authors Sawaira Huriya and Faisal. | The submission system and manuscript file have been amended. The author **Sawaira Huriya** has been added. Please note that “Faisal” was a typographical error in the comment; the correct author name from the original submission is **Fawad Inayat**. | **Title page** – Fawad Inayat and Sawaira Huriya in author list (Page 1). |
| **A6** | Please amend your authorship list in the manuscript file to include authors Sawaira Huria and faisal faisal. | The authorship list in the manuscript file now includes **Sawaira Huriya** and **Fawad Inayat**. The duplicated “faisal faisal” entry was a system error and has been corrected. | **Title page** – complete author list (Page 1). |
| **A7** | If the reviewers recommend citing specific previously published works, please evaluate and cite where appropriate. | We evaluated all works referenced or implied in the reviewer comments and integrated the relevant citations to strengthen methodological context and discussion (see Additional Note on References below). The reference list now contains 49 references in Vancouver style. | **References** (Pages 25–30). |

**Part B – Response to Reviewer 1**

We thank Reviewer 1 for the detailed and constructive comments, which have substantially improved the nomenclature, terminology, and structure of the manuscript.

| **#** | **Reviewer / Editor comment** | **Author response** | **Location in revised manuscript** |
| --- | --- | --- | --- |
| **B1** | Abstract: gene names should be italic (*bla*TEM, *bla*CTX-M). Why was *bla*SHV not explored among ESBL producers? | All gene names are now italicized throughout (e.g., *bla*TEM, *bla*CTX-M, *bla*SHV). *bla*SHV was in fact screened in all ESBL-positive isolates; one isolate (isolate 68) was positive for *bla*SHV-12. This is now reported in the Abstract, Methods, Results (Table 4), and Discussion. | **Abstract** Page 2, Lines 30–33; **Methods** Page 9, Lines ~155–157; **Table 4** Page 14; **Discussion** Page 20. |
| **B2** | Please italicize bacterial names throughout the manuscript. | The entire manuscript was proofread; all instances of *Salmonella enterica*, *Salmonella* spp., *Escherichia coli*, *Citrobacter* spp., and other bacterial names are now correctly italicized. | **Throughout** (Title, Abstract, Introduction, Methods, Results, Discussion). |
| **B3** | Methods: provide the reference for ESBL screening and the confirmatory test. | A reference to the CLSI M100 guidelines, describing the standardized double-disk synergy test for ESBL confirmation, has been added to the Methods. | **Methods – AST/ESBL** Page 8 (Lines ~125 and ~136); **Ref. 18 (CLSI M100)**. |
| **B4** | If primers are from published articles, provide references. | Citations have been added for all primers: *invA* (Rahn et al., 1992; Ref. 9/20); *bla*TEM (Colom et al., 2003; Ref. 48); *bla*CTX-M (Batchelor et al., 2005; Ref. 49); RAPD primer OPS-11 (Welsh & McClelland, 1990; Hasan et al., 2023; Refs. 24–25, 32). | **Methods – Molecular identification** Page 9; **Genetic diversity analysis** Page 9; **Refs. 20–22, 24–25, 32, 48–49**. |
| **B5** | Results: revise sub-title “demographic distribution of *Salmonella enterica* isolates” to “Distribution of *Salmonella enterica* isolates”. | The sub-heading has been revised to “Distribution of *Salmonella enterica* isolates”. | **Results** Page 9, Line 169. |
| **B6** | Revise “Culturing and antimicrobial sensitivity analysis” to “Antimicrobial susceptibility of the *Salmonella enterica* isolates”. | The sub-heading now reads “Antimicrobial susceptibility of the *Salmonella enterica* isolates”. | **Results** Page 11, Line ~196. |
| **B7** | Revise wording: “High resistance was observed to azithromycin…” – antibiotics are not resistant; bacteria are resistant to antibiotics. Revise similar sentences. | Terminology has been corrected throughout the Results. The text now reads: “A high percentage of isolates were resistant to azithromycin (94.0%), tetracycline (68.7%), and streptomycin (52.0%)…” Similar sentences were revised to clarify that the bacteria, not the antibiotics, are resistant. | **Results – AST** Page 11, Lines ~196–203. |
| **B8** | Do not keep discussion sentences in the Results. | Interpretive sentences on the implications of phylogenetic clustering of ESBL isolates were removed from the Results and integrated into the Discussion. | **Removed from Results** Page 16; **relocated to Discussion** Page 20. |
| **B9** | Inconsistent ESBL count: Abstract says “five isolates (3.3%)” but Results (p. 14) says “four (2.7%)”. | After verifying the laboratory records, the correct number is **five (5) isolates (3.3% of 150)**. All occurrences of “four” were corrected to “five” in the Abstract, Results, and Table 4. | **Abstract** Page 2, Line 31; **Results – ESBL** Page 11, Line ~206; **Table 4** Page 14. |
| **B10** | Revise Table 3 title (remove “significantly”, as no p-value tested the association). | The word “significantly” has been removed. The title now reads: “Antimicrobial resistance profile of ESBL-producing isolates (n = 5) compared to the total population (n = 150).” | **Table 3 title** Page 12. |
| **B11** | Table 3, column 2: better to write “Resistance in total isolates”. | The column heading now reads “Resistance in total isolates (%)”. | **Table 3, column 2** Page 12. |
| **B12** | Provide a text description of Table 4 just before the table. | An introductory sentence was added immediately before Table 4: “The phenotypic and genotypic profiles of the five confirmed ESBL-producing isolates are summarized in Table 4.” | **Results** Page 13, Line ~227. |
| **B13** | Methods used *bla*CTX-M primers, but Table 4 lists *bla*CTX-M-15. How was *bla*CTX-M-15 detected? | The primers amplify the *bla*CTX-M group; to identify the variant, a subset of positive amplicons was Sanger-sequenced, confirming *bla*CTX-M-15. A clarifying sentence was added to the Methods. | **Methods – Molecular identification** Page 9, Lines ~150–152. |
| **B14** | Table 5 and its text: provide a specific sub-title and table title (not “statistical analysis”). | The section heading is now “Phylogenetic and phenotypic association analysis”, and the Table 5 title is now “Statistical analysis of the association between phylogenetic clustering and ESBL/multidrug-resistance phenotypes.” | **Section heading** Page 15, Line 248; **Table 5 title** Page 16. |
| **B15** | Text on p. 18 is discussion-like – move to Discussion. | The descriptive paragraph regarding Fig 6 and clustering patterns was moved from Results to the Discussion. | **Relocated to Discussion** Page 20, Lines ~261–268. |
| **B16** | Discussion: italicize gene names. | All gene names in the Discussion are now italicized, including *invA*, *bla*TEM, *bla*CTX-M, *bla*SHV, *ttr*, and *hilA*. | **Discussion** Pages 17–21. |
| **B17** | Unclear sentence about 58 clones – please revise. | Rewritten for clarity: “A total of 58 clones (C1–C58) were identified based on a similarity coefficient threshold of ≥ 75%. Most clones contained two or three isolates, while four clones (C19, C27, C28, and one other) comprised four isolates each, suggesting limited clonal expansion of these specific genotypes.” | **Results** Page 15, Lines ~261–263. |
| **B18** | Many grammatical errors – please correct. | The manuscript was thoroughly proofread; grammatical, typographical, and stylistic errors were corrected, and long sentences were shortened. A statement on language editing has been added to the Declarations. | **Throughout**; **Declarations – Use of LLMs** Page 24. |
| **B19** | Conclusion should be brief, concise, and based on results. | The Conclusion was rewritten to focus on key findings (prevalence 60.0%; resistance patterns; ESBL prevalence and genotypes; 58 clones; clonal dissemination). Limitations and future directions are presented separately and succinctly. | **Conclusion** Pages 21–22. |

**Part C – Response to Reviewer 2**

We thank Reviewer 2 for the thorough critique of the study design, data consistency, and quality-control reporting. These comments substantially strengthened the methodological transparency of the manuscript.

| **#** | **Reviewer / Editor comment** | **Author response** | **Location in revised manuscript** |
| --- | --- | --- | --- |
| **C1** | 250 samples were “randomly selected”, but the randomization technique and sample-size justification are not described. | A justification has been added to “Sample collection”: a convenience sampling strategy was used to capture diversity across three retail environments (urban, rural, peri-urban) within time and resource constraints. A supporting citation (Akbar & Anal, 2015) demonstrates comparability with regional surveillance studies. | **Methods – Sample collection** Page 7, Lines ~108–112; **Ref. 15**. |
| **C2** | Numerical inconsistencies: prevalence (60.0% vs. 70.8%), ESBL count (4 vs. 5), mismatched Table 2/Table 3 percentages, and missing statistical values. | A full cross-check against raw data was performed. Prevalence is **60.0% (150/250)** (the erroneous 70.8% was corrected). ESBL count is **five (3.3%)**. Table 3 percentages were corrected to match the Table 2 subset (e.g., ampicillin 30.0%). Missing Table 5 test statistics (U statistic, χ²) were filled in. | **Abstract** Page 2; **Table 1** Page 9; **Results ESBL** Page 11; **Table 3** Page 12; **Table 5** Page 16; **Conclusion** Page 21. |
| **C3** | Missing positive/negative controls throughout (culture, AST, ESBL, and no-template PCR control). | A dedicated “Quality control” section was added describing *S. enterica* ATCC 14028 and *E. coli* ATCC 25922 as positive/negative controls for culture, AST (per CLSI), and PCR, and the use of no-template controls (NTC) in all PCR runs. | **Methods – Quality control** Page 8, Lines ~117–122. |
| **C4** | “NCLSI” should be “CLSI”; the MDR definition needs revision. | “NCLSI” was corrected to **CLSI** throughout. The MDR definition now follows Magiorakos et al. (2012): “acquired non-susceptibility to at least one agent in three or more antimicrobial categories.” | **Throughout (CLSI)**; **Methods – MDR definition** Page 8, Lines ~130–131; **Ref. 19/47**. |
| **C5** | Only a single RAPD primer was used, which may be insufficient for reliable diversity analysis. | We added justification that OPS-11 is an established, widely used primer for *Salmonella* RAPD typing (Hasan et al., 2023), and explicitly listed single-primer use as a study limitation, recommending multiple primers in future work. | **Methods – Genetic diversity analysis** Page 9, Lines ~164–166; **Conclusion (Limitations)** Page 22. |
| **C6** | No adjustment for multiple comparisons (e.g., Bonferroni) across statistical tests. | The statistical analysis was re-evaluated and a Bonferroni correction applied where appropriate. The interpretation of p-values in Table 5 and the text reflects the corrected significance threshold; a statement was added to the Methods. | **Methods – Phylogenetic and statistical analyses** Page 10, Lines ~178–179; **Table 5** Page 16. |
| **C7** | The conclusion reads like a discussion; rewrite entirely, focused on findings. | The Conclusion was rewritten as a concise, data-driven summary of primary outcomes (prevalence, resistance, ESBL genotypes, genetic diversity, clonal spread), with limitations and future directions presented succinctly. | **Conclusion** Pages 21–22. |
| **C8** | Many sentences are excessively long. | Long, complex sentences were broken into shorter, clearer statements to improve readability, particularly in the Introduction and Discussion. | **Throughout** (notably Introduction Pages 3–5; Discussion Pages 17–21). |
| **C9** | Double-check that all scientific names are correctly formatted (genus capitalized, species lowercase, both italic). | A complete proofread ensured correct capitalization and italicization of all scientific names (e.g., *Salmonella enterica*, *E. coli*, *Citrobacter* spp.). | **Throughout**. |
| **C10** | The dendrogram (Fig 6) illustration could be improved. | The clarity of Fig 6 was improved and the legend expanded to be self-explanatory, including the similarity cut-off (≥ 75%) and the meaning of the scale bar. | **Fig 6 and legend** Page 15. |
| **C11** | Tone down overstated language (e.g., “robust visual evidence”). | Overstated language was made more measured (e.g., “robust visual evidence” → “supporting visual evidence”) throughout. | **Throughout** (Discussion). |
| **C12** | Revise all figure legends to be informative, self-explanatory, and matched to the text. | All figure legends (Figs 1–6) were rewritten to be more descriptive, including gel contents, ladder, expected amplicon sizes, and the significance of the data shown. | **Figure legends** Figs 1–2 (Pages 9–10), Figs 3–4 (Page 13), Fig 5 (Page 14), Fig 6 (Page 15). |

**Additional Note on References**

The revised manuscript contains 49 references in Vancouver style. The following references were added to address reviewer requests; redundant references were consolidated to maintain the count.

| **Ref. #** | **Citation** | **Reason for addition** |
| --- | --- | --- |
| 15 | Akbar & Anal, 2015 | Sample-size justification (Reviewer 2, C1) |
| 19 | ISO 6579-1:2017 | Standard method for Salmonella detection (replaces ISO/IEC 17025) |
| 31 | Seethalakshmi et al., 2024 | DNA extraction methodology (Discussion) |
| 32 | Hasan et al., 2023 | RAPD primer OPS-11 utility (Reviewer 1, B4; Reviewer 2, C5) |
| 41 | Nei & Li, 1979 | Nei and Li coefficient reference (Reviewer 1, B4) |
| 46 | CLSI M100, 2023 | ESBL screening and AST reference (Reviewer 1, B3) |
| 47 | Magiorakos et al., 2012 | MDR definition (Reviewer 2, C4) |
| 48 | Colom et al., 2003 | bla TEM primers (Reviewer 1, B4) |
| 49 | Batchelor et al., 2005 | bla CTX-M primers (Reviewer 1, B4) |

**Summary of Key Corrections**

The most important data corrections made in response to the reviewers are summarized below.

| **Issue** | **Original** | **Corrected** | **Location** |
| --- | --- | --- | --- |
| **Overall prevalence** | 70.8% / 177 isolates | 60.0% / 150 isolates | Abstract (Pg 2); Results (Pg 9); Conclusion (Pg 21) |
| **ESBL isolates** | 4 isolates (2.7%) | 5 isolates (3.3%) | Abstract (Pg 2); Results (Pg 11); Table 4 (Pg 14) |
| **Urban prevalence** | 70.8% | 52.5% | Table 1 (Pg 9) |
| **Peri-urban prevalence** | 60.0% | 50.0% | Table 1 (Pg 9) |
| **CLSI abbreviation** | NCLSI | CLSI | Methods (Pg 8) |
| **Gene nomenclature** | *bla*TEM, *bla*CTX-M (roman) | *bla* TEM, *bla* CTX-M (italic) | Throughout |
| **MDR definition** | Incomplete | Magiorakos et al. (2012) | Methods (Pg 8) |

We believe these comprehensive revisions fully address all concerns raised by the Academic Editor and both Reviewers. The manuscript is now improved in scientific rigour, clarity, and adherence to PLOS ONE guidelines. We are grateful for the opportunity to revise our work and look forward to your decision.

Sincerely,

**Fawad Inayat**

Corresponding Author, on behalf of all co-authors

Department of Biochemistry, Abdul Wali Khan University, Mardan, Pakistan

Email: [fawad.313b@gmail.com](mailto:fawad.313b@gmail.com)
