# Supplementary material for: Phenotypic and molecular characterization of Salmonella enterica isolated from retail beef in Peshawar, Pakistan
Source: PLoS One. 2026 Jul 29;21(7):e0352859. doi: 10.1371/journal.pone.0352859 (PMC13419216; doi:10.1371/journal.pone.0352859)
Supplement: Peer review history

# Author comment: Author response to Decision Letter 0

**DOI:** 10.1371/journal.pone.0352859.r001

**Transfer Alert**

This paper was transferred from another journal. As a result, its full editorial history (including decision letters, peer reviews and author responses) may not be present.

9 Jan 2026

# Peer review: Decision Letter 0

**DOI:** 10.1371/journal.pone.0352859.r002

**Authors:** Dwij Bhatta

11 Apr 2026

PONE-D-26-01381

PHENOTYPIC AND MOLECULAR DIVERSITY ANALYSIS OF SALMONELLA ENTERICA CONTAMINATING BEEF SAMPLES SOLD IN PESHAWAR, PAKISTAN

PLOS One

Dear Dr. Inayat,

Thank you for submitting your manuscript to PLOS ONE. After careful consideration, we feel that it has merit but does not fully meet PLOS ONE’s publication criteria as it currently stands. Therefore, we invite you to submit a revised version of the manuscript that addresses the points raised during the review process.

Please submit your revised manuscript by May 26 2026 11:59PM. If you will need more time than this to complete your revisions, please reply to this message or contact the journal office at plosone@plos.org. When you're ready to submit your revision, log on to https://www.editorialmanager.com/pone/ and select the 'Submissions Needing Revision' folder to locate your manuscript file.

Please include the following items when submitting your revised manuscript:

- A letter that responds to each point raised by the academic editor and reviewer(s). You should upload this letter as a separate file labeled 'Response to Reviewers'.
- A marked-up copy of your manuscript that highlights changes made to the original version. You should upload this as a separate file labeled 'Revised Manuscript with Track Changes'.
- An unmarked version of your revised paper without tracked changes. You should upload this as a separate file labeled 'Manuscript'.

If you would like to make changes to your financial disclosure, please include your updated statement in your cover letter. Guidelines for resubmitting your figure files are available below the reviewer comments at the end of this letter.

If applicable, we recommend that you deposit your laboratory protocols in protocols.io to enhance the reproducibility of your results. Protocols.io assigns your protocol its own identifier (DOI) so that it can be cited independently in the future. For instructions see: https://journals.plos.org/plosone/s/submission-guidelines#loc-laboratory-protocols. Additionally, PLOS ONE offers an option for publishing peer-reviewed Lab Protocol articles, which describe protocols hosted on protocols.io. Read more information on sharing protocols at https://plos.org/protocols?utm_medium=editorial-email&utm_source=authorletters&utm_campaign=protocols.

As the corresponding author, your ORCID iD is verified in the submission system and will appear in the published article. PLOS supports the use of ORCID, and we encourage all coauthors to register for an ORCID iD and use it as well. Please encourage your coauthors to verify their ORCID iD within the submission system before final acceptance, as unverified ORCID iDs will not appear in the published article. *Only*  the individual author can complete the verification step; PLOS staff *cannot*  verify ORCID iDs on behalf of authors.

We look forward to receiving your revised manuscript.

Kind regards,

Dwij Raj Bhatta, PhD

Academic Editor

PLOS One

Journal Requirements:

When submitting your revision, we need you to address these additional requirements.

1. Please ensure that your manuscript meets PLOS ONE's style requirements, including those for file naming. The PLOS ONE style templates can be found at

https://journals.plos.org/plosone/s/file?id=wjVg/PLOSOne_formatting_sample_main_body.pdf and

https://journals.plos.org/plosone/s/file?id=ba62/PLOSOne_formatting_sample_title_authors_affiliations.pdf

2. PLOS ONE now requires that authors provide the original uncropped and unadjusted images underlying all blot or gel results reported in a submission’s figures or Supporting Information files. This policy and the journal’s other requirements for blot/gel reporting and figure preparation are described in detail at https://journals.plos.org/plosone/s/figures#loc-blot-and-gel-reporting-requirements and https://journals.plos.org/plosone/s/figures#loc-preparing-figures-from-image-files. When you submit your revised manuscript, please ensure that your figures adhere fully to these guidelines and provide the original underlying images for all blot or gel data reported in your submission. See the following link for instructions on providing the original image data: https://journals.plos.org/plosone/s/figures#loc-original-images-for-blots-and-gels.

In your cover letter, please note whether your blot/gel image data are in Supporting Information or posted at a public data repository, provide the repository URL if relevant, and provide specific details as to which raw blot/gel images, if any, are not available. Email us at plosone@plos.org if you have any questions.

3. Thank you for stating the following in the Competing Interests section:

no competing interest

Please confirm that this does not alter your adherence to all PLOS ONE policies on sharing data and materials, by including the following statement: "This does not alter our adherence to PLOS ONE policies on sharing data and materials.” (as detailed online in our guide for authors http://journals.plos.org/plosone/s/competing-interests). If there are restrictions on sharing of data and/or materials, please state these. Please note that we cannot proceed with consideration of your article until this information has been declared.

Please include your updated Competing Interests statement in your cover letter; we will change the online submission form on your behalf.

4. In the online submission form, you indicated that will be available from the corresponding author

All PLOS journals now require all data underlying the findings described in their manuscript to be freely available to other researchers, either a. In a public repository, b. Within the manuscript itself, or c. Uploaded as supplementary information.

This policy applies to all data except where public deposition would breach compliance with the protocol approved by your research ethics board. If your data cannot be made publicly available for ethical or legal reasons (e.g., public availability would compromise patient privacy), please explain your reasons on resubmission and your exemption request will be escalated for approval.

5. Please amend the manuscript submission data (via Edit Submission) to include authors Sawaira Huriya and Faisal

6. Please amend your authorship list in your manuscript file to include authors Sawaira Huria and faisal faisal

7. If the reviewer comments include a recommendation to cite specific previously published works, please review and evaluate these publications to determine whether they are relevant and should be cited. There is no requirement to cite these works unless the editor has indicated otherwise.

Additional Editor Comments:

The athors need to revise manuscript by following the reviewers queries and comments!

[Note: HTML markup is below. Please do not edit.]

Reviewers' comments:

Reviewer's Responses to Questions

**Comments to the Author**

1. Is the manuscript technically sound, and do the data support the conclusions?

The manuscript must describe a technically sound piece of scientific research with data that supports the conclusions. Experiments must have been conducted rigorously, with appropriate controls, replication, and sample sizes. The conclusions must be drawn appropriately based on the data presented.

Reviewer #1: Partly

Reviewer #2: Partly

**********

2. Has the statistical analysis been performed appropriately and rigorously?

Reviewer #1: Yes

Reviewer #2: N/A

**********

3. Have the authors made all data underlying the findings in their manuscript fully available?

The PLOS Data policy requires authors to make all data underlying the findings described in their manuscript fully available without restriction, with rare exception (please refer to the Data Availability Statement in the manuscript PDF file). The data should be provided as part of the manuscript or its supporting information, or deposited to a public repository. For example, in addition to summary statistics, the data points behind means, medians and variance measures should be available. If there are restrictions on publicly sharing data—e.g. participant privacy or use of data from a third party—those must be specified.

Reviewer #1: Yes

Reviewer #2: No

**********

4. Is the manuscript presented in an intelligible fashion and written in standard English?

PLOS ONE does not copyedit accepted manuscripts, so the language in submitted articles must be clear, correct, and unambiguous. Any typographical or grammatical errors should be corrected at revision, so please note any specific errors here.

Reviewer #1: No

Reviewer #2: Yes

**********

5. Review Comments to the Author

Please use the space provided to explain your answers to the questions above. You may also include additional comments for the author, including concerns about dual publication, research ethics, or publication ethics. (Please upload your review as an attachment if it exceeds 20,000 characters)

Reviewer #1: Title of the manuscript: Phenotypic and molecular diversity analysis of Salmonella enterica contaminating beef samples sold in Peshawar, Pakistan

The authors described the diversity of Salmonella enterica from beef samples sold in Peshawar, Pakistan. This manuscript is of interest for limited readerships. It has many places where improvement can be done. I recommend for major revision of the manuscript.

1. Abstract, the names of genes should be written as blaTEM, blaCTX-M (italic). Why did you not explore blaSHV among ESBL producer?

2. Please make the name of bacteria italic throughout the manuscript.

3. Methods, please provide the reference for ESBL screening and confirmatory test.

4. If you use the primers from already published article, please provide the reference.

5. Results, please revise the sub-title, “demographic distribution of Salmonella enterica isolates”. You can just say “Distribution of Salmonella enterica isolates”.

6. The sub-title “Culturing and antimicrobial sensitivity analysis” should be revised. You can write “Antimicrobial susceptibility of the Salmonella enterica isolates”.

7. The text description of Table 2 should be revised. “High resistance was observed to azithromycin (94.0%), tetracycline (68.7%), and streptomycin (52.0%), indicating widespread multidrug resistance”. In fact, it is not high resistance. It means “High percentage of isolates were resistant to azithromycin (94.0%), tetracycline (68.7%), and streptomycin (52.0%).” Similarly, antibiotics are not resistant. Bacteria is resistant to antibiotics. Please revise other sentences in the same paragraph.

8. Please do not keep discussion sentences in the results.

9. In results of abstract, it is mentioned that “Five isolates (3.3%) were confirmed as extended-spectrum ß-lactamase (ESBL) producers…………..” but in results in page 14, it has been mentioned as “Among the 150 Salmonella enterica isolates, four (2.7%) were confirmed as extended-spectrum ß-lactamase (ESBL) producers …………..”. In following sentences, there is also five ESBL producers.

10. Please revise the title of Table 3 “ESBL-producing isolates (n=5) exhibited significantly higher resistance rates compared to the overall population (n=150).” Further, you have not used p-value to test the significant association. Therefore, please delete “significantly”.

11. Table 3, column 2, better to write “Resistance in total isolates”.

12. Please provide the text description of Table 4, just before the table.

13. In methods, it has been mentioned that blaCTX-M gene specific primers were used. But in Table 4, it has given blaCTX-M-15. How did you detect blaCTX-M-15?

14. Table 5 and its text, please revise the sub-title and title of the Table 5. Please provide specific sub-title and title of the Table 5. It cannot be statistical analysis.

15. The text description of Page 18 is related to discussion. Please shift this paragraph to discussion.

16. Discussion, please make the name of genes italic.

17. “The results of this study, Showed that there were 58 clones, out of which 4 clones have maximum isolates of four and mostly were grouped in two or three isolates.” Not clear. Please revise.

18. There are many grammatical errors in the manuscript. Please correct those errors.

19. Conclusion should be brief, concise and based on results. Please keep only essentials in the conclusion.

Reviewer #2: Study conducted by Ahmad et al. entitled “Phenotypic and Molecular Diversity Analysis of Salmonella enterica Contaminating Beef Samples Sold in Peshawar, Pakistan” addresses the prevalence, genetic diversity, and antimicrobial resistance (including ESBL production) of Salmonella enterica contaminating beef sold in local markets of Peshawar, Pakistan. Overall, this study may address an important public health issue in a regional setting in Pakistan. However, standard scientific writing, many typographical errors, several methodological issues, including statistical analysis, and presentation issues (in results, conclusion), need to be carefully addressed.

Major Points:

1.Authors mentioned 250 samples were randomly selected, however, do not describe the randomization technique (the authors provide no justification or calculation for how this number was determined), this may raise question about whether the study was adequately powered to detect meaningful differences.

2.Several calculations and numerical data in the results section are inconsistent or incomplete, including discrepancies in prevalence reporting (60.0% vs. 70.8%), contradictions in the number of ESBL-producing isolates (4 vs. 5), mismatched resistance percentages between Table 2 and Table 3, and missing statistical values and figures. For example, the prevalence of Salmonella enterica is reported as 60.0% in the abstract, results text, and Table 1, but incorrectly rises to 70.8% in the conclusion section.

3.Missing positive and negative controls throughout

It's surprising that no control strains are mentioned anywhere—not for culture, not for AST, not for ESBL confirmation, and not even a no-template control for PCR. This makes it hard to get convinced with the results.

4.Authors have written 'NCLSI', I think authors mean 'CLSI' (or possibly the old name 'NCCLS'). Please fix that. Also, definition of MDR isn't quite right and needs to be revised.

5.Only a single RAPD primer was used, which may not be sufficient for reliable genetic diversity analysis.

6.No adjustment for multiple comparisons (e.g., Bonferroni) was applied across several statistical tests.

7.The conclusion reads more like a discussion than a proper conclusion. It wanders away from the main findings, which is a problem. A conclusion should be concise and clearly reflect the key findings from the data, but that's missing here. The authors need to rewrite this section entirely, keeping it focused on what the study actually found.

Minor points

The list of required minor revisions may be quite long. To name just a few examples:

1.Throughout the manuscript, many sentences are excessively long and contain multiple independent clauses. This syntactical complexity impedes readability and distracts the reader from the core findings. To improve clarity and flow, the authors should revise the text to use shorter, simpler sentences that each convey a single, coherent, and contextual idea.

2.I would recommend that the authors go through the whole manuscript and double-check that all scientific names are formatted correctly. Remember: genus capitalized, species epithet lowercase, and both italicized. A lot of mistakes are found throughout the manuscript.

3.Illustration of dendrogram (Figure 6) could be improved.

4.The authors should tone down some of the language throughout the manuscript. For example, phrases like "robust visual evidence" are overstatements. Please use more measured, objective wording.

5.Please revise all figure legends to be more informative and self-explanatory, ensuring they accurately match the corresponding figures as described in the text.

**********

6. PLOS authors have the option to publish the peer review history of their article (what does this mean?). If published, this will include your full peer review and any attached files.

If you choose “no”, your identity will remain anonymous but your review may still be made public.

**Do you want your identity to be public for this peer review?**  For information about this choice, including consent withdrawal, please see our Privacy Policy.

Reviewer #1: No

Reviewer #2: No

**********

[NOTE: If reviewer comments were submitted as an attachment file, they will be attached to this email and accessible via the submission site. Please log into your account, locate the manuscript record, and check for the action link "View Attachments". If this link does not appear, there are no attachment files.]

To ensure your figures meet our technical requirements, please review our figure guidelines: https://journals.plos.org/plosone/s/figures

You may also use PLOS’s free figure tool, NAAS, to help you prepare publication quality figures: https://journals.plos.org/plosone/s/figures#loc-tools-for-figure-preparation.

NAAS will assess whether your figures meet our technical requirements by comparing each figure against our figure specifications.

Attachment Submitted filename: PONE-D-26-0-1381.docx

# Author comment: Author response to Decision Letter 1

**DOI:** 10.1371/journal.pone.0352859.r003

14 Jun 2026

Date: 31 May 2026

To:

Dr. Dwij Raj Bhatta

Academic Editor, PLOS ONE

Re: Response to Reviewers – Manuscript PONE-D-26-01381 (Revised)

Title: Phenotypic and Molecular Characterization of Salmonella enterica Isolated from Retail Beef in Peshawar, Pakistan

Dear Dr. Bhatta,

We sincerely thank you and the reviewers for the time and effort invested in evaluating our manuscript. The comments were constructive and have substantially improved the clarity, rigour, and presentation of our work. We have carefully revised the manuscript to address every point raised by the editorial office and both reviewers.

Below we provide a detailed, point-by-point response to each comment. For every revision, we indicate the page number and line number in the revised manuscript file (“Revised_Manuscript_Highlighted.docx”), in which all changes are marked with yellow highlighting to facilitate verification. Line numbers refer to the continuous line numbering applied throughout the revised manuscript. A clean (unhighlighted) version is also provided. This response is organized into three parts:

• Part A – Response to Journal Requirements (Academic Editor)

• Part B – Response to Reviewer 1

• Part C – Response to Reviewer 2

We trust that the revisions fully address all concerns and that the manuscript now meets the publication standards of PLOS ONE. The page and line references below correspond to the revised manuscript file as submitted.

Part A – Response to Journal Requirements (Academic Editor)

# Reviewer / Editor comment Author response Location in revised manuscript

A1 Please ensure that your manuscript meets PLOS ONE’s style requirements, including those for file naming. The manuscript has been fully reformatted to PLOS ONE style: Times New Roman 12 pt, double line spacing, continuous line numbering, page numbering, justified body text, PLOS-style figure labels (“Fig 1”–“Fig 6”) and table labels, and Vancouver-style references. Files are named according to journal conventions (Revised_Manuscript, Revised_Manuscript_Highlighted, Response_to_Reviewers). Entire manuscript (formatting applied throughout).

A2 PLOS ONE requires the original uncropped and unadjusted images underlying all blot or gel results. Original, uncropped, and unadjusted gel images for Figs 1–5 have been prepared and will be uploaded as Supporting Information (S1 Raw Images) at resubmission. All figures comply with the journal’s guidance on cropping, contrast adjustment, and labelling, as confirmed in the cover letter. Noted in cover letter; S1 Raw Images uploaded separately.

A3 Please confirm that your Competing Interests statement does not alter your adherence to PLOS ONE policies on sharing data and materials. The Competing Interests statement now reads: “The authors have declared that no competing interests exist. This does not alter our adherence to PLOS ONE policies on sharing data and materials.” Declarations – Competing interests (Page 24).

A4 All data underlying the findings must be freely available without restriction. All underlying datasets have been deposited in the Zenodo public repository and are freely available. The Data Availability Statement now includes the DOI link (https://zenodo.org/records/18700727; DOI: 10.5281/zenodo.18700727). Declarations – Availability of data and materials (Page 24).

A5 Please amend the submission data to include authors Sawaira Huriya and Faisal. The submission system and manuscript file have been amended. The author Sawaira Huriya has been added. Please note that “Faisal” was a typographical error in the comment; the correct author name from the original submission is Fawad Inayat. Title page – Fawad Inayat and Sawaira Huriya in author list (Page 1).

A6 Please amend your authorship list in the manuscript file to include authors Sawaira Huria and faisal faisal. The authorship list in the manuscript file now includes Sawaira Huriya and Fawad Inayat. The duplicated “faisal faisal” entry was a system error and has been corrected. Title page – complete author list (Page 1).

A7 If the reviewers recommend citing specific previously published works, please evaluate and cite where appropriate. We evaluated all works referenced or implied in the reviewer comments and integrated the relevant citations to strengthen methodological context and discussion (see Additional Note on References below). The reference list now contains 49 references in Vancouver style. References (Pages 25–30).

Part B – Response to Reviewer 1

We thank Reviewer 1 for the detailed and constructive comments, which have substantially improved the nomenclature, terminology, and structure of the manuscript.

# Reviewer / Editor comment Author response Location in revised manuscript

B1 Abstract: gene names should be italic (blaTEM, blaCTX-M). Why was blaSHV not explored among ESBL producers? All gene names are now italicized throughout (e.g., blaTEM, blaCTX-M, blaSHV). blaSHV was in fact screened in all ESBL-positive isolates; one isolate (isolate 68) was positive for blaSHV-12. This is now reported in the Abstract, Methods, Results (Table 4), and Discussion. Abstract Page 2, Lines 30–33; Methods Page 9, Lines ~155–157; Table 4 Page 14; Discussion Page 20.

B2 Please italicize bacterial names throughout the manuscript. The entire manuscript was proofread; all instances of Salmonella enterica, Salmonella spp., Escherichia coli, Citrobacter spp., and other bacterial names are now correctly italicized. Throughout (Title, Abstract, Introduction, Methods, Results, Discussion).

B3 Methods: provide the reference for ESBL screening and the confirmatory test. A reference to the CLSI M100 guidelines, describing the standardized double-disk synergy test for ESBL confirmation, has been added to the Methods. Methods – AST/ESBL Page 8 (Lines ~125 and ~136); Ref. 18 (CLSI M100).

B4 If primers are from published articles, provide references. Citations have been added for all primers: invA (Rahn et al., 1992; Ref. 9/20); blaTEM (Colom et al., 2003; Ref. 48); blaCTX-M (Batchelor et al., 2005; Ref. 49); RAPD primer OPS-11 (Welsh & McClelland, 1990; Hasan et al., 2023; Refs. 24–25, 32). Methods – Molecular identification Page 9; Genetic diversity analysis Page 9; Refs. 20–22, 24–25, 32, 48–49.

B5 Results: revise sub-title “demographic distribution of Salmonella enterica isolates” to “Distribution of Salmonella enterica isolates”. The sub-heading has been revised to “Distribution of Salmonella enterica isolates”. Results Page 9, Line 169.

B6 Revise “Culturing and antimicrobial sensitivity analysis” to “Antimicrobial susceptibility of the Salmonella enterica isolates”. The sub-heading now reads “Antimicrobial susceptibility of the Salmonella enterica isolates”. Results Page 11, Line ~196.

B7 Revise wording: “High resistance was observed to azithromycin…” – antibiotics are not resistant; bacteria are resistant to antibiotics. Revise similar sentences. Terminology has been corrected throughout the Results. The text now reads: “A high percentage of isolates were resistant to azithromycin (94.0%), tetracycline (68.7%), and streptomycin (52.0%)…” Similar sentences were revised to clarify that the bacteria, not the antibiotics, are resistant. Results – AST Page 11, Lines ~196–203.

B8 Do not keep discussion sentences in the Results. Interpretive sentences on the implications of phylogenetic clustering of ESBL isolates were removed from the Results and integrated into the Discussion. Removed from Results Page 16; relocated to Discussion Page 20.

B9 Inconsistent ESBL count: Abstract says “five isolates (3.3%)” but Results (p. 14) says “four (2.7%)”. After verifying the laboratory records, the correct number is five (5) isolates (3.3% of 150). All occurrences of “four” were corrected to “five” in the Abstract, Results, and Table 4. Abstract Page 2, Line 31; Results – ESBL Page 11, Line ~206; Table 4 Page 14.

B10 Revise Table 3 title (remove “significantly”, as no p-value tested the association). The word “significantly” has been removed. The title now reads: “Antimicrobial resistance profile of ESBL-producing isolates (n = 5) compared to the total population (n = 150).” Table 3 title Page 12.

B11 Table 3, column 2: better to write “Resistance in total isolates”. The column heading now reads “Resistance in total isolates (%)”. Table 3, column 2 Page 12.

B12 Provide a text description of Table 4 just before the table. An introductory sentence was added immediately before Table 4: “The phenotypic and genotypic profiles of the five confirmed ESBL-producing isolates are summarized in Table 4.” Results Page 13, Line ~227.

B13 Methods used blaCTX-M primers, but Table 4 lists blaCTX-M-15. How was blaCTX-M-15 detected? The primers amplify the blaCTX-M group; to identify the variant, a subset of positive amplicons was Sanger-sequenced, confirming blaCTX-M-15. A clarifying sentence was added to the Methods. Methods – Molecular identification Page 9, Lines ~150–152.

B14 Table 5 and its text: provide a specific sub-title and table title (not “statistical analysis”). The section heading is now “Phylogenetic and phenotypic association analysis”, and the Table 5 title is now “Statistical analysis of the association between phylogenetic clustering and ESBL/multidrug-resistance phenotypes.” Section heading Page 15, Line 248; Table 5 title Page 16.

B15 Text on p. 18 is discussion-like – move to Discussion. The descriptive paragraph regarding Fig 6 and clustering patterns was moved from Results to the Discussion. Relocated to Discussion Page 20, Lines ~261–268.

B16 Discussion: italicize gene names. All gene names in the Discussion are now italicized, including invA, blaTEM, blaCTX-M, blaSHV, ttr, and hilA. Discussion Pages 17–21.

B17 Unclear sentence about 58 clones – please revise. Rewritten for clarity: “A total of 58 clones (C1–C58) were identified based on a similarity coefficient threshold of ≥ 75%. Most clones contained two or three isolates, while four clones (C19, C27, C28, and one other) comprised four isolates each, suggesting limited clonal expansion of these specific genotypes.” Results Page 15, Lines ~261–263.

B18 Many grammatical errors – please correct. The manuscript was thoroughly proofread; grammatical, typographical, and stylistic errors were corrected, and long sentences were shortened. A statement on language editing has been added to the Declarations. Throughout; Declarations – Use of LLMs Page 24.

B19 Conclusion should be brief, concise, and based on results. The Conclusion was rewritten to focus on key findings (prevalence 60.0%; resistance patterns; ESBL prevalence and genotypes; 58 clones; clonal dissemination). Limitations and future directions are presented separately and succinctly. Conclusion Pages 21–22.

Part C – Response to Reviewer 2

We thank Reviewer 2 for the thorough critique of the study design, data consistency, and quality-control reporting. These comments substantially strengthened the methodological transparency of the manuscript.

# Reviewer / Editor comment Author response Location in revised manuscript

C1 250 samples were “randomly selected”, but the randomization technique and sample-size justification are not described. A justification has been added to “Sample collection”: a convenience sampling strategy was used to capture diversity across three retail environments (urban, rural, peri-urban) within time and resource constraints. A supporting citation (Akbar & Anal, 2015) demonstrates comparability with regional surveillance studies. Methods – Sample collection Page 7, Lines ~108–112; Ref. 15.

C2 Numerical inconsistencies: prevalence (60.0% vs. 70.8%), ESBL count (4 vs. 5), mismatched Table 2/Table 3 percentages, and missing statistical values. A full cross-check against raw data was performed. Prevalence is 60.0% (150/250) (the erroneous 70.8% was corrected). ESBL count is five (3.3%). Table 3 percentages were corrected to match the Table 2 subset (e.g., ampicillin 30.0%). Missing Table 5 test statistics (U statistic, χ²) were filled in. Abstract Page 2; Table 1 Page 9; Results ESBL Page 11; Table 3 Page 12; Table 5 Page 16; Conclusion Page 21.

C3 Missing positive/negative controls throughout (culture, AST, ESBL, and no-template PCR control). A dedicated “Quality control” section was added describing S. enterica ATCC 14028 and E. coli ATCC 25922 as positive/negative controls for culture, AST (per CLSI), and PCR, and the use of no-template controls (NTC) in all PCR runs. Methods – Quality control Page 8, Lines ~117–122.

C4 “NCLSI” should be “CLSI”; the MDR definition needs revision. “NCLSI” was corrected to CLSI throughout. The MDR definition now follows Magiorakos et al. (2012): “acquired non-susceptibility to at least one agent in three or more antimicrobial categories.” Throughout (CLSI); Methods – MDR definition Page 8, Lines ~130–131; Ref. 19/47.

C5 Only a single RAPD primer was used, which may be insufficient for reliable diversity analysis. We added justification that OPS-11 is an established, widely used primer for Salmonella RAPD typing (Hasan et al., 2023), and explicitly listed single-primer use as a study limitation, recommending multiple primers in future work. Methods – Genetic diversity analysis Page 9, Lines ~164–166; Conclusion (Limitations) Page 22.

C6 No adjustment for multiple comparisons (e.g., Bonferroni) across statistical tests. The statistical analysis was re-evaluated and a Bonferroni correction applied where appropriate. The interpretation of p-values in Table 5 and the text reflects the corrected significance threshold; a statement was added to the Methods. Methods – Phylogenetic and statistical analyses Page 10, Lines ~178–179; Table 5 Page 16.

C7 The conclusion reads like a discussion; rewrite entirely, focused on findings. The Conclusion was rewritten as a concise, data-driven summary of primary outcomes (prevalence, resistance, ESBL genotypes, genetic diversity, clonal spread), with limitations and future directions presented succinctly. Conclusion Pages 21–22.

C8 Many sentences are excessively long. Long, complex sentences were broken into shorter, clearer statements to improve readability, particularly in the Introduction and Discussion. Throughout (notably Introduction Pages 3–5; Discussion Pages 17–21).

C9 Double-check that all scientific names are correctly formatted (genus capitalized, species lowercase, both italic). A complete proofread ensured correct capitalization and italicization of all scientific names (e.g., Salmonella enterica, E. coli, Citrobacter spp.). Throughout.

C10 The dendrogram (Fig 6) illustration could be improved. The clarity of Fig 6 was improved and the legend expanded to be self-explanatory, including the similarity cut-off (≥ 75%) and the meaning of the scale bar. Fig 6 and legend Page 15.

C11 Tone down overstated language (e.g., “robust visual evidence”). Overstated language was made more measured (e.g., “robust visual evidence” → “supporting visual evidence”) throughout. Throughout (Discussion).

C12 Revise all figure legends to be informative, self-explanatory, and matched to the text. All figure legends (Figs 1–6) were rewritten to be more descriptive, including gel contents, ladder, expected amplicon sizes, and the significance of the data shown. Figure legends Figs 1–2 (Pages 9–10), Figs 3–4 (Page 13), Fig 5 (Page 14), Fig 6 (Page 15).

Additional Note on References

The revised manuscript contains 49 references in Vancouver style. The following references were added to address reviewer requests; redundant references were consolidated to maintain the count.

Ref. # Citation Reason for addition

15 Akbar & Anal, 2015 Sample-size justification (Reviewer 2, C1)

19 ISO 6579-1:2017 Standard method for Salmonella detection (replaces ISO/IEC 17025)

31 Seethalakshmi et al., 2024 DNA extraction methodology (Discussion)

32 Hasan et al., 2023 RAPD primer OPS-11 utility (Reviewer 1, B4; Reviewer 2, C5)

41 Nei & Li, 1979 Nei and Li coefficient reference (Reviewer 1, B4)

46 CLSI M100, 2023 ESBL screening and AST reference (Reviewer 1, B3)

47 Magiorakos et al., 2012 MDR definition (Reviewer 2, C4)

48 Colom et al., 2003 bla TEM primers (Reviewer 1, B4)

49 Batchelor et al., 2005 bla CTX-M primers (Reviewer 1, B4)

Summary of Key Corrections

The most important data corrections made in response to the r

Attachment Submitted filename: Response_to_Reviewers.docx

# Editor report: Decision Letter 1

**DOI:** 10.1371/journal.pone.0352859.r004

**Authors:** Dwij Bhatta

16 Jun 2026

Phenotypic and Molecular Characterization of Salmonella enterica Isolated from Retail Beef in Peshawar, Pakistan

PONE-D-26-01381R1

Dear Dr. Inayat,

We’re pleased to inform you that your manuscript has been judged scientifically suitable for publication and will be formally accepted for publication once it meets all outstanding technical requirements.

Within one week, you’ll receive an e-mail detailing the required amendments. When these have been addressed, you’ll receive a formal acceptance letter and your manuscript will be scheduled for publication.

An invoice will be generated when your article is formally accepted. Please note, if your institution has a publishing partnership with PLOS and your article meets the relevant criteria, all or part of your publication costs will be covered. Please make sure your user information is up-to-date by logging into Editorial Manager at Editorial Manager® and clicking the ‘Update My Information' link at the top of the page. For questions related to billing, please contact billing support.

If your institution or institutions have a press office, please notify them about your upcoming paper to help maximize its impact. If they’ll be preparing press materials, please inform our press team as soon as possible -- no later than 48 hours after receiving the formal acceptance. Your manuscript will remain under strict press embargo until 2 pm Eastern Time on the date of publication. For more information, please contact onepress@plos.org.

Kind regards,

Dwij Raj Bhatta, PhD

Academic Editor

PLOS One

Additional Editor Comments (optional):

Authors have significantly revised &improved manuscript as per reviewers comments,now it can be accepted for publication.

Reviewers' comments:

# Editor report: Acceptance letter

**DOI:** 10.1371/journal.pone.0352859.r005

**Authors:** Dwij Bhatta

PONE-D-26-01381R1

PLOS One

Dear Dr. Inayat,

I'm pleased to inform you that your manuscript has been deemed suitable for publication in PLOS One. Congratulations! Your manuscript is now being handed over to our production team.

At this stage, our production department will prepare your paper for publication. This includes ensuring the following:

* All references, tables, and figures are properly cited

* All relevant supporting information is included in the manuscript submission,

* There are no issues that prevent the paper from being properly typeset

You will receive further instructions from the production team, including instructions on how to review your proof when it is ready. Please keep in mind that we are working through a large volume of accepted articles, so please give us a few days to review your paper and let you know the next and final steps.

Lastly, if your institution or institutions have a press office, please let them know about your upcoming paper now to help maximize its impact. If they'll be preparing press materials, please inform our press team within the next 48 hours. Your manuscript will remain under strict press embargo until 2 pm Eastern Time on the date of publication. For more information, please contact onepress@plos.org.

You will receive an invoice from PLOS for your publication fee after your manuscript has reached the completed accept phase. If you receive an email requesting payment before acceptance or for any other service, this may be a phishing scheme. Learn how to identify phishing emails and protect your accounts at https://explore.plos.org/phishing.

If we can help with anything else, please email us at customercare@plos.org.

Thank you for submitting your work to PLOS ONE and supporting open access.

Kind regards,

PLOS ONE Editorial Office Staff

on behalf of

Professor Dwij Raj Bhatta

Academic Editor

PLOS One
